# In Vitro Metabolism and Transport Characteristics of Zastaprazan

**DOI:** 10.3390/pharmaceutics16060799

**Published:** 2024-06-13

**Authors:** Min Seo Lee, Jihoon Lee, Minyoung Pang, John Kim, Hyunju Cha, Banyoon Cheon, Min-Koo Choi, Im-Sook Song, Hye Suk Lee

**Affiliations:** 1 College of Pharmacy and BK21 Four-Sponsored Advanced Program for SmartPharma Leaders, The Catholic University of Korea, Bucheon 14662, Republic of Korea; minseo.lee@catholic.ac.kr; 2 BK21 FOUR Community-Based Intelligent Novel Drug Discovery Education Unit, Vessel-Organ Interaction Research Center (VOICE), Research Institute of Pharmaceutical Sciences, College of Pharmacy, Kyungpook National University, Daegu 41566, Republic of Korea; legadema0905@knu.ac.kr; 3 College of Pharmacy, Dankook University, Cheonan 30019, Republic of Korea; mignon@dankook.ac.kr (M.P.); minkoochoi@dankook.ac.kr (M.-K.C.); 4 Onconic Therapeutics Inc., Seoul 06236, Republic of Korea; john.kim@onconic.co.kr (J.K.); hj.cha@onconic.co.kr (H.C.); bycheon@onconic.co.kr (B.C.)

**Keywords:** zastaprazan (JP-1366), potassium-competitive acid blocker (P-CAB), metabolite identification, drug-metabolizing enzymes, drug transporters

## Abstract

Zastaprazan (JP-1366), a novel potassium-competitive acid blocker, is a new drug for the treatment of erosive esophagitis. JP-1366 is highly metabolized in human, mouse, and dog hepatocytes but moderately metabolized in rat and monkey hepatocytes when estimated from the metabolic stability of this compound in hepatocyte suspension and when 18 phase I metabolites and 5 phase II metabolites [i.e., *N*-dearylation (M6), hydroxylation (M1, M19, M21), dihydroxylation (M7, M8, M14, M22), trihydroxylation (M13, M18), hydroxylation and reduction (M20), dihydroxylation and reduction (M9, M16), hydrolysis (M23), hydroxylation and glucuronidation (M11, M15), hydroxylation and sulfation (M17), dihydroxylation and sulfation (M10, M12), *N*-dearylation and hydroxylation (M3, M4), *N*-dearylation and dihydroxylation (M5), and *N*-dearylation and trihydroxylation (M2)] were identified from JP-1366 incubation with the hepatocytes from humans, mice, rats, dogs, and monkeys. Based on the cytochrome P450 (CYP) screening test and immune-inhibition analysis with CYP antibodies, CYP3A4 and CYP3A5 played major roles in the metabolism of JP-1366 to M1, M3, M4, M6, M8, M9, M13, M14, M16, M18, M19, M21, and M22. CYP1A2, 2C8, 2C9, 2C19, and 2D6 played minor roles in the metabolism of JP-1366. UDP-glucuronosyltransferase (UGT) 2B7 and UGT2B17 were responsible for the glucuronidation of M1 to M15. However, JP-1366 and active metabolite M1 were not substrates for drug transporters such as organic cation transporter (OCT) 1/2, organic anion transporter (OAT) 1/3, organic anion transporting polypeptide (OATP)1B1/1B3, multidrug and toxic compound extrusion (MATE)1/2K, P-glycoprotein (P-gp), and breast cancer-resistant protein (BCRP). Only M1 showed substrate specificity for P-gp. The findings indicated that drug-metabolizing enzymes, particularly CYP3A4/3A5, may have a significant role in determining the pharmacokinetics of zastaprazan while drug transporters may only have a small impact on the absorption, distribution, and excretion of this compound.

## 1. Introduction

Diseases associated with gastric acid, such as gastroesophageal reflux disease (GERD) and peptic ulcer disease, involve typical symptoms such as heartburn and acid reflux, most of which are chronic and highly likely to recur, resulting in long-term pain [1]. The global prevalence of GERD is 13.98% with regional variability. The incidence rate of GERD is high in North America at 19.55% and about 12.88% ~ 14.12% in Asia, Europe, Latin America, and Oceania [2].

Therapeutics to cure or alleviate symptoms by suppressing or removing gastric acid secretion have been used. Proton-pump inhibitor (PPI) drugs are activated in an acidic environment and irreversibly bind to the proton pump (H^+^/K^+^-ATPase) to inhibit gastric acid secretion [3]. Therefore, esomeprazole, the most frequently used PPI, requires an acidic environment for structural transformation and takes about 3 to 5 days to exert a sufficient effect [4]. In addition, it has been observed that esomeprazole exhibits adequate efficacy for nocturnal acid secretion, although it suffers from intersubject therapeutic differences caused by pharmacogenetic issues on cytochrome P450 (CYP) 2C19. Because CYP2C19 is the major metabolizing enzyme for PPIs and their metabolites are pharmacologically inactive, extensive metabolizers with severe erosive esophagitis demonstrated a very low (16.7%) healing rate [5]. Poor metabolizers with a CYP2C19 mutation showed a five-fold higher plasma exposure to omeprazole [5].

Potassium-competitive acid blockers (P-CABs) are novel antisecretory drugs that bind to the α subunit of H^+^/K^+^-ATPase and compete with potassium ions [6,7]. P-CABs bind with the proton pump by forming a reversible ionic bond rather than a covalent bond that necessitates acid activation [1,7]. Additionally, P-CABs have a relatively longer half-life compared to PPIs [8]. Due to these characteristics, P-CABs are a superior therapy option to PPIs since they function more quickly and maintain their antiacid secretory effect throughout the whole day [9]. In 2007, the first-in-class P-CAB anti-ulcer drug Revanex (revaprazan) was released in Korea. Since then, P-CAB drugs have been developed; however, many of them have been discontinued due to inadequate efficacy or dose-dependent hepatotoxicity [10]. The currently available P-CABs on the market are revaprazan, vonoprazan, tegoprazan, and fexuprazan [10,11,12].

Zastaprazan (JP-1366), a novel P-CAB, was approved for the treatment of erosive esophagitis under the brand name JAQBO by the Ministry of Food and Drug Safety (MFDS) of the Republic of Korea on 24 April 2024, and is undergoing the preparation of documents for market approval by the USA’s FDA and in other countries. It was safe and well tolerated after a single-ascending oral dose (SAD) of up to 60 mg and a multiple-ascending oral dose (MAD) of up to 40 mg in healthy male Korean subjects with no clinically significant safety or tolerability assessments, including hepatotoxicity [1]. The plasma concentration and pharmacokinetics parameters, including C_max_ and AUC of JP-1366, showed dose proportionality in all doses of SAD (5 mg–60 mg) and MAD (5 mg–40 mg) in the study, and the mean accumulation ratio was 1.17–1.79 [1]. In addition, the correlation between the systemic exposure of JP-1366 and the response (the percentage of time that the gastric pH was over 4.0; % time pH > 4.0) was well explained by the sigmoid E_max_ model [1]. In a comparative pharmacodynamics study, the % time pH > 4.0 over a period of 24 h was significantly higher in the JP-1366 40 mg dose group (91.84% ± 4.06% for SAD; 89.28% ± 9.12% for MAD) compared to those of the esomeprazole 40 mg dose group (72.06% ± 13.43% for SAD; 56.56% ± 22.47% for MAD), which indicated that there was a stronger acid suppression in JP-1366 than that of esomeprazole at nighttime [1].

In the present study, the metabolism of JP-1366 was compared among human, dog, monkey, mouse, and rat hepatocytes, and the possible in vitro metabolites of JP-1366 formed from the hepatocyte incubation were elucidated. We also explained the drug-metabolizing enzymes responsible for the metabolism of JP-1366 (i.e., CYP and UDP-glucuronosyltransferase (UGT)) and investigated substrate specificity for clinically important drug transporters (i.e., organic cation transporter (OCT) 1/2, organic anion transporter (OAT) 1/3, organic anion transporting polypeptide (OATP)1B1/1B3, multidrug and toxic compound extrusion (MATE)1/2K, P-glycoprotein (P-gp), and breast cancer-resistant protein (BCRP)) [13,14,15] to understand the contribution of drug-metabolizing enzymes and transporters to the pharmacokinetics of JP-1366.

## 2. Materials and Methods

### 2.1. Materials

Zastaprazan, (JP-1366, (1-azetidinyl(8-(((2,6-dimethylphenyl) methyl)amino)-2,3-dimethylimidazo(1,2-a)pyridin-6-yl)methanone), purity, 100%); M1 (azetidin-1-yl(8-((2-(hydroxymethyl)-6-methyl-benzyl)amino)-2,3-dimethylimidazo[1,2-a]pyridin-6-yl)methanone, purity, 97.59%); M6 ((8-amino-2,3-dimethylimidazo[1,2-a]pyridin-6-yl)(azetidin-1-yl)methanone, purity, 99.56%); and M23 (8-((2,6-dimethylbenzyl)amino)-2,3-dimethylimidazo[1,2-a]pyridine-6-carboxylic acid, purity, 98.78%) were provided by Onconic Therapeutics (Seoul, Republic of Korea). Glucose-6-phosphate sodium salt, β-nicotinamide adenine dinucleotide phosphate (NADP^+^), reduced β-nicotinamide adenine dinucleotide phosphate tetrasodium salt (NADPH), glucose-6-phosphate dehydrogenase, uridine 5′-diphosphoglucuronic acid trisodium salt (UDPGA), alamethicin from trichoderma viride, lansoprazole, cimetidine, cyclosporine A (CsA), Ko134 hydrate, probenecid, rifampin, and sodium butyrate were purchased from Sigma-Aldrich Co. (St. Louis, MO, USA). SN-38 glucuronide was obtained from Toronto Research Chemicals Inc. (Toronto, ON, Canada). Penicillin/streptomycin, gentamicin, and hygromycin B were purchased from Invitrogen (Waltham, MA, USA).

Cryopreserved male rat and mouse hepatocytes, opti-incubate hepatocyte media, and OptiThaw cryohepatocyte kit were purchased from XenoTech (Kansas City, KS, USA). LiverPool 50 donor human hepatocytes and INVITROGRO HT Medium were obtained from BIOIVT (Hicksville, NY, USA). Cryopreserved male dog and monkey hepatocytes; UltraPool human liver microsomes; human cDNA-expressed CYPs supersomes; human cDNA-expressed UGTs supersomes; HEK293-mock cells; HEK293 cells overexpressing individual drug transporters (i.e., MATE1/2K, OCT1/2, OAT1/3, and OATP1B1/1B3 transporters); LLC-PK1-P-gp (LLC-PK1 cells stably expressing P-gp); phosphate-buffered saline; Dulbecco’s Modified Eagle’s Medium (DMEM); Medium 199; fetal bovine serum (FBS); and human CYP1A2-, 2C8-, 2D6-, or 3A4-selective antibodies (i.e., anti-CYP1A2, anti-CYP2C8, anti-CYP2D6, or anti-CYP3A4) were purchased from Corning Life Sciences (Woburn, MA, USA). LLC-PK1-BCRP (LLC-PK1 cells stably expressing BCRP) was obtained from Dr. A.H. Schinkel at Netherlands Cancer Institute (Amsterdam, The Netherlands). Caco-2 cells were purchased from American Type Culture Collection (Manassas, VA, USA). Hank’s balanced salt solution (HBSS) was purchased from Welgene (Gyeongsan, Republic of Korea). Deionized water, acetonitrile, and methanol were of LC-MS grade and obtained from Fisher Scientific Co. (Pittsburgh, PA, USA).

### 2.2. Metabolic Stability of JP-1366 in Human, Mouse, Rat, Dog, and Monkey Hepatocytes

According to the manufacturer’s protocol, cryopreserved human, dog, monkey, mouse, and rat hepatocytes were purified and recovered. Aliquots (60 μL) of hepatocyte suspension at a final density of 1.0 × 10^6^ cells/mL and 60 μL of 1 μM JP-1366 were added in a 96-well plate, gently mixed, and incubated for 5, 10, 15, 20, 30, 45, 60, or 90 min in a CO_2_ incubator. The metabolic reaction in each sample well was quenched by adding 120 μL of ice-cold acetonitrile. After sonification for 10 min and centrifugation of the reaction mixture (13,000 rpm, 10 min, 4 °C), aliquots (20 μL) of the supernatant were mixed with 180 μL of 50% acetonitrile solution containing 50 ng/mL SN-38-glucuronide (internal standard, IS) and an aliquot (5 μL) and processed for the analysis of JP-1366.

### 2.3. In Vitro Metabolic Profiling of JP-1366 in Hepatocytes

Cryopreserved human, dog, monkey, mouse, and rat hepatocytes were purified and recovered according to the manufacturer’s protocol. Aliquots (60 μL) of hepatocyte suspension at a final density of 1.0 × 10^6^ cells/mL and 60 μL of 10 μM JP-1366 were added in a 96-well plate, gently mixed, and incubated for 60 min in a CO_2_ incubator. The metabolic reaction in each sample well was quenched by adding 120 μL of ice-cold acetonitrile. After sonification for 10 min and centrifugation of the reaction mixture (13,000 rpm, 10 min, 4 °C), aliquots (200 μL) of the supernatant were evaporated under a N_2_ stream followed by reconstitution with 10% acetonitrile (100 μL) and processed to identify the possible metabolites of JP-1366.

### 2.4. Screening of CYP and UGT Isozymes Involved in JP-1366 Metabolism

The experimental protocols were performed in accordance with FDA guidelines and the previous reports with slight modifications [13,16]. To investigate the inhibitory effect of CYP inhibitor, SKF-525A on JP-1366 metabolism, the reaction mixtures containing 80 μL of 50 mM potassium phosphate buffer (pH 7.4), 4 μL of 250 mM magnesium chloride, 1 μL of 500 μM JP-1366 (final concentration: 5 μM), 1 μL of 1 mM or 10 mM SKF-525A (final concentration: 10 μM and 100 μM), 10 μL of 2 mg/mL pooled human liver microsomes (final concentration: 0.2 mg/mL), and 5 μL of NADPH-generating system were incubated at 37 °C for 30 min. The reactions were stopped by adding 100 μL of lansoprazole (IS) in ice-cold acetonitrile (100 ng/mL). After the centrifugation of the reaction mixture (13,000 rpm, 5 min, 4 °C), aliquots (20 μL) of the supernatant were diluted with 180 μL of 10% acetonitrile and processed to analyze JP-1366 metabolites. To investigate the involvement of esterase in the metabolism of JP-1366, 1 μL of 10 mM or 100 mM NaF (final concentration: 100 μM and 1000 μM) was added to the reaction mixture instead of SKF-525A.

To characterize the CYP enzymes responsible for the metabolism of JP-1366 and its metabolites, M1 and M6, the reaction mixture consisted of 80 μL of 50 mM potassium phosphate buffer (pH 7.4), 4 μL of 250 mM magnesium chloride, 1 μL of 500 μM JP-1366 (M1 or M6, final concentration: 5 μM), and 10 μL of 0.4 μM human cDNA-expressed isozymes (CYPs 1A2, 2A6, 2B6, 2C8, 2C9, 2C19, 2D6, 2E1, 3A4, and 3A5) (final concentration, 4 pmol). The reaction was initiated by adding 5 μL of NADPH-generating system (1 mM NADPH, 5 mM glucose 6-phosphate, 1 mM NADP^+^, and 1.0 U glucose 6-phosphate dehydrogenase) in triplicate, and the mixture was further incubated for 30 min at 37 °C in a shaking water bath. The reaction was terminated by adding 100 μL of lansoprazole (IS) in ice-cold acetonitrile (100 ng/mL). After the centrifugation of the reaction mixture (13,000 rpm, 5 min, 4 °C), aliquots (20 μL) of the supernatant were diluted with 180 μL of 10% acetonitrile and processed to analyze JP-1366 metabolites.

To characterize UGT isozymes responsible for the glucuronidation of M1, the reaction mixture consisted of 79 μL of 50 mM Tris buffer (pH 7.4), 4 μL of 250 mM magnesium chloride, 1 μL of 1000 μM M1 (final concentration: 10 μM), 1 μL of 2.5 mg/mL alamethicin, 10 μL of 2 mg/mL pooled human liver microsomes (final concentration: 0.2 mg/mL), or human cDNA-expressed UGT enzymes (UGT 1A1, 1A3, 1A4, 1A6, 1A7, 1A8, 1A9, 1A10, 2B7, 2B17) (final concentration: 0.1 mg/mL protein). The reaction was initiated by adding 5 μL of UDPGA (final concentration: 5 mM), and the mixture was further incubated for 30 min at 37 °C in a shaking water bath. The reaction was terminated by adding 100 μL of lansoprazole (IS) in ice-cold acetonitrile (100 ng/mL). After the centrifugation of the reaction mixture (13,000 rpm, 5 min, 4 °C), aliquots (20 μL) of the supernatant were diluted with 180 μL of 10% acetonitrile and processed to analyze JP-1366 metabolites.

### 2.5. Enzyme Kinetics of JP-1366 Metabolism in Human Liver Microsomes, CYP, and UGT Enzymes

To evaluate the enzyme kinetic parameters for the metabolism of JP-1366, the concentration-dependent metabolism of JP-1366 and M1 was conducted according to the previous reports [16]. Briefly, the reaction mixture consisted of 80 μL of 50 mM potassium phosphate buffer (pH 7.4); 4 μL of 250 mM magnesium chloride; 10 μL of 2 mg/mL pooled human liver microsomes (final concentration: 0.2 mg/mL) or human cDNA-expressed CYP1A2, 2C8, 2C9, 2C19, 2D6, 3A4, and 3A5 enzymes (final concentration: 4 pmol); and 1 μL of various concentrations of JP-1366 (final concentration: 1, 2, 5, 10, 20, 40, 60, or 80 μM). The reaction was initiated by adding 5 μL of NADPH-generating system in triplicate, and the mixture was further incubated for 30 min at 37 °C in a shaking water bath. The reaction was terminated by adding 100 μL of lansoprazole (IS) in ice-cold acetonitrile (500 ng/mL). After the centrifugation of the reaction mixture (13,000 rpm, 5 min, 4 °C), aliquots (20 μL) of the supernatant were diluted with 380 μL of 10% acetonitrile and processed to analyze JP-1366 metabolites.

To investigate the enzyme kinetics for the glucuronidation of M1, the reaction mixture consisted of 79 μL of 50 mM Tris buffer (pH 7.4), 4 μL of 250 mM magnesium chloride, 1 μL of various concentrations of M1 (final concentration: 2, 5, 10, 20, 40, 60, 80, or 100 μM), 1 μL of 2.5 mg/mL alamethicin, 10 μL of 2 mg/mL pooled human liver microsomes (final concentration: 0.2 mg/mL), or human cDNA-expressed UGT enzymes (UGT 2B7 and 2B17) (final concentration: 0.1 mg/mL protein). By adding 5 μL of UDPGA (final concentration, 5 mM), the reaction was initiated, and the mixture was further incubated for 30 min at 37 °C in a shaking water bath. The reaction was terminated by adding 100 μL of ice-cold acetonitrile containing 5 ng/mL of lansoprazole (IS). After the centrifugation of the reaction mixture (13,000 rpm, 5 min, 4 °C), aliquots (20 μL) of the supernatant were diluted with 80 μL of 50% acetonitrile and processed to analyze JP-1366 metabolites.

### 2.6. Immuno-Inhibition of JP-1366 Metabolism with CYP Antibodies

The use of antibodies specific to individual CYP can be used to confirm the contribution of individual CYP in the metabolism of drugs or drug candidates according to the FDA guidelines [13]. To increase the selectivity of antibodies to a single CYP, we chose monoclonal antibodies, which bind to a single epitope of CYP and are treated with ultrapooled HLMs according to the previous reports in addition to the manufacturer’s protocols [16,17,18,19].

Immuno-inhibition experiments were conducted by incubating human liver microsomes with various amounts of human CYP1A2-, 2C8-, 2D6-, or 3A4-selective antibodies (i.e., anti-CYP1A2, anti-CYP2C8, anti-CYP2D6, or anti-CYP3A4) for 15 min on ice. The reaction was initiated by adding 70 μL of 50 mM potassium phosphate buffer (pH 7.4), 1 μL of JP-1366 (final concentration: 5 μM), 4 μL of 250 mM magnesium chloride, and 5 μL of NADPH-generating system, and the mixture was further incubated for 15 min at 37 °C in a shaking water bath. The reaction was terminated by adding 100 μL of lansoprazole (IS) in ice-cold acetonitrile (100 ng/mL). After the centrifugation of the reaction mixture (13,000 rpm, 5 min, 4 °C), aliquots (20 μL) of the supernatant were diluted with 180 μL of 10% acetonitrile and processed to analyze JP-1366 metabolites.

### 2.7. Uptake of JP-1366 and M1 in HEK293 Cells Overexpressing Drug Transporters

HEK293-mock and HEK293 cells overexpressing MATE1/2K, OCT1/2, OAT1/3, and OATP1B1/1B3 transporters were maintained with 8% CO_2_/92% air at 37 °C in DMEM supplemented with 10% FBS and 5 mM non-essential amino acids. In the case of OATP1B1/1B3 and MATE1/2K cells, 2 mM of sodium butyrate was added to the culture medium to improve transport activity [20]. Cells were seeded at 2 × 10^5^ cells/well in poly-D-lysine-coated 24-well plates. Growth medium was discarded after 24 h, and attached cells were washed with HBSS and preincubated for 20 min in HBSS at 37 °C. Stock solutions of JP1366 or M1 and representative inhibitors (cimetidine, probenecid, and rifampin) were diluted in HBSS to make a final concentration of 100 μM of cimetidine (for MATE1/2K and OCT1/2), 20 μM of probenecid (for OAT1/3), and 20 μM of rifampin (for OATP1B1/1B3) [21,22]. Uptake of 6 μM JP-1366 or M1 was measured in the absence and presence of representative inhibitors for 5 min at 37 °C. Plates were immediately placed on ice, and cells were then washed twice with 1 mL of ice-cold HBSS. Residual HBSS was removed thoroughly from the plates. Subsequently, 150 μL of 80% ice-cold acetonitrile containing 150 ng/mL of lansoprazole (IS) was added to each sample well, and the cell plates were shaken gently for 20 min at 4 °C. Aliquots (20 μL) of the supernatant were diluted with 80 μL of 50% acetonitrile, and an aliquot (5 μL) was injected onto the LC-MS/MS system.

### 2.8. Permeability of JP-1366 and M1

Caco-2 cells (passage no. 41–42) were grown in tissue culture flasks containing Dulbecco’s Modified Eagle Medium supplemented with 20% FBS, 1% nonessential amino acids, and 1% penicillin–streptomycin. Caco-2 cells were seeded on collagen-coated 24-transwell membranes at a density of 2.5 × 10^5^ cells/mL and maintained at 37 °C in a humidified atmosphere with 5% CO_2_/95% air for 21 days. The culture medium was replaced every other day. On the day of the experiment, the growth medium was discarded, and the attached cells were washed with prewarmed HBSS (pH 7.4) and preincubated with HBSS for 20 min at 37 °C. To measure the apical-to-basal permeability (P_app,AB_), 0.3 mL of HBSS containing 4 µM of JP1366 or M1 was added to the apical side (inside of the insert), and 0.7 mL of fresh HBSS was added to the basal side of the insert. To measure the basal-to-apical permeability (P_app,BA_), 0.7 mL of HBSS containing 4 µM of JP-1366 or M1 was added to the basal side, and 0.3 mL of fresh HBSS was added to the apical side of the insert. Aliquots (0.1 mL) in the apical side were transferred to clean tubes every 15 min for 1 h and replaced with an equal volume of fresh HBSS. Aliquots (30 μL) of each sample were vortex-mixed with 90 μL of IS solution for 1 min, and then centrifugation was performed at 13,500 rpm at 4 °C for 5 min. Aliquots (5 μL) of the supernatant were injected into the LC-MS/MS for analysis. Using the previous method by Jeon et al., permeability marker compounds such as propranolol (for high permeability) and atenolol (for low permeability) were used for the comparison of their Caco-2 permeability with JP-1366 or M1 [23,24].

The involvement of P-gp and BCRP in the efflux of JP-1366 or M1 was investigated in LLC-PK1 cells overexpressing P-gp or BCRP. LLC-PK1-P-gp or BCRP cells were seeded on collagen-coated 24-transwell membranes at a density of 2.5 × 10^5^ cells/mL and maintained at 37 °C in a humidified atmosphere with 5% CO_2_/95% air for 5 days in Medium 199 supplemented with 10% fecal bovine serum, 50 μg/mL of gentamycin, and 50 μg/mL of hygromycin. The culture medium was replaced every other day. On the day of the experiment, the growth medium was discarded, and the attached cells were washed with prewarmed HBSS (pH 7.4) and preincubated with HBSS for 20 min at 37 °C. For P_app,AB_, 0.3 mL of HBSS containing 4 µM of JP1366 or M1 in the presence or absence of inhibitor (20 mM CsA for P-gp; 1 mM Ko134 for BCRP) was added to the apical side, and 0.7 mL of fresh HBSS was added to the basal side of the insert. For P_app,BA_, 0.7 mL of HBSS containing 4 µM of JP1366 or M1 in the presence or absence of inhibitor (20 mM CsA for P-gp; 1 mM Ko134 for BCRP) was added to the basal side, and 0.3 mL of fresh HBSS was added to the apical side of the insert. Aliquots (0.1 mL) in the apical side were transferred to clean tubes every 15 min for 1 h and replaced with an equal volume of fresh HBSS.

The concentration dependency in the P_app,AB_ and P_app,BA_ of JP-1366 in LLC-PK1-P-gp and BCRP cells was measured. LLC-PK1-P-gp or BCRP cells were seeded on collagen-coated 24-transwell membranes at a density of 2.5 × 10^5^ cells/mL and cultured for 5 days. The growth medium was discarded on the day of the experiment, and the attached cells were washed with prewarmed HBSS (pH 7.4) and preincubated with HBSS for 20 min at 37 °C. For P_app,AB_, 0.3 mL of HBSS containing various concentrations of JP-1366 (0.4–100 µM) or M1 (0.3–85 µM) was added to the apical side, and 0.7 mL of fresh HBSS was added to the basal side of the insert. For P_app,BA_, 0.7 mL of HBSS containing various concentrations of JP-1366 (0.4–100 µM) or M1 (0.3–85 µM) was added to the basal side, and 0.3 mL of fresh HBSS was added to the apical side of the insert. Aliquots (0.1 mL) in the apical side were transferred to clean tubes every 15 min for 1 h and replaced with an equal volume of fresh HBSS. Aliquots (30 μL) of each sample were vortex-mixed with 90 μL of IS solution for 1 min, and then centrifugation was performed at 13,500 rpm at 4 °C for 5 min. Aliquots (5 μL) of the supernatant were injected into the LC-MS/MS for analysis.

### 2.9. LC-MS/MS Analysis of JP-1366 and Its Metabolites

For the identification of JP-1366 metabolites, Nexera X2 (Shimadzu, Kyoto, Japan) LC coupled with a Q-Exactive Orbitrap mass spectrometer (Thermo Scientific Inc., Waltham, MA, USA) was used. Analytes were separated in ACQUITY UPLC HSS T3 (1.8 μm, 100 × 2.1 mm) (Waters, Milford, MA, USA), which exhibited excellent peak shape, better separation, and good sensitivity for JP-1366 and its metabolites among tested columns. Gradient elution condition was also optimized using 0.1% formic acid in 5% acetonitrile (mobile phase A) and 0.1% formic acid in 95% acetonitrile (mobile phase B) to obtain successfully separated and reasonable peak shapes for JP-1366 and its metabolites. The optimized gradient elution condition for the profiling of JP-1366 metabolites was as follows: 4% B for 0–0.5 min, 4–30% B for 0.5–35 min, 30–95% B for 35–35.1 min, 95% B held for 35.1–40 min, 95–4% B for 40–40.1 min, and 4% B for 40.1–45 min with a flow rate of 0.4 mL/min. MS spectra were obtained in positive-ion mode using electrospray ionization under optimized conditions as follows: capillary temperature, 350 °C; sheath gas flow rate, 40 (arbitrary units); aux gas flow rate, 10 (arbitrary units); and aux gas heater temperature, 350 °C. Full MS scan with data-dependent MS/MS was performed from *m*/*z* 100 to *m*/*z* 1000.

An Agilent 1290 Infinity LC coupled to a 6495 triple quadrupole MS (Agilent Technologies, Wilmington, DE, USA) was used for the quantification of JP-1366 and its metabolites. Samples were separated in ACQUITY UPLC HSS T3 (1.8 μm, 100 × 2.1 mm) (Waters, MA, USA) and the optimized gradient elution condition and a flow rate was as follows: 4% B for 0–0.5 min, 4–30% B for 0.5–35 min, 30–95% B for 35–35.1 min, 95% B held for 35.1–40 min, 95–4% B for 40–40.1 min, and 4% B for 40.1–45 min with a flow rate of 0.4 mL/min for reaction phenotyping analyses; 5% B for 0–1 min, 5–98% B for 1–3 min, 98% B held for 3–6 min, 98–5% B for 6–6.1 min, and 5% B for 6.1–9 min with a flow rate of 0.4 mL/min for metabolic stability, cell uptake, and permeability studies. For the metabolic stability study of JP-1366 and M1, selective reaction monitoring transitions were *m*/*z* 363.1 → 119.1 for JP-1366 and *m*/*z* 569.0 → 392.9 for SN-38-Glucuronide (IS) at collision energies of 20 and 10 eV, respectively. Table 1 presents the mass condition that was applied for reaction phenotyping analyses.

The analytical method for the simultaneous determination of JP-1366, M1, and M6 was validated in the linearity, accuracy, precision, matrix effect, and post-preparation stability. The calibration curves for the metabolic stability, cell uptake, and permeability studies were linear at concentration ranges of 0.2–150 pmol for JP-1366 and 0.48–60 pmol for M1, with correlation coefficients of determination (r^2^) ≥ 0.9950. The intra-day accuracy and precision at the low-, middle-, and high-quality controls (QCs) (3.6, 15, and 135 pmol for JP-1366 and 1.44, 6, and 54 pmol for M1) were 88.4–104.8% with 1.8–3.9% of the coefficient of variance (CV). The calibration curves for the reaction phenotyping analyses were linear at concentration ranges of 0.5–500 pmol for JP-1366, 1–1000 pmol for M1, and 2–1000 pmol for M6, with r^2^ ranging from 0.9916 to 0.9996. The intra- and inter-day accuracy and precision at the low, middle, and high QCs were 92.6–113.0% with 4.3–13.5% CV, and the post-preparation stability was 92.8–107.1% with 3.3–7.6% CV. Matrix effects of JP-1366 and M1 in human liver microsomes ranged from 94.0–114.5% with 0.6–1.8% CV.

### 2.10. Data Analysis and Statistical Analysis

Log-transformed relative responses of JP-1366 were plotted against incubation time, thereby obtaining the elimination slope (k) from the linear regression analysis. Elimination parameters from JP-1366 metabolic stability were calculated using the following equations [25].

(1) $$t_{\frac{1\;}{2}}(min)=\frac{ln2}{k\left(elimination\;slope\right)}$$

(2) $${Cl}_{int}(mL/min/kg)=\frac{ln2}{t_{\frac{1}{2}}}\times \frac{mL\;incubation}{hepatocyte\;(10^{6}\;cells)}\times \frac{B\times 10^{6}\;cells}{g\;liver}\times \frac{A\;g\;liver}{kg\;BW}$$

(3) $${Cl}_{hep}(mL/min/kg)=\frac{Q_{h}\times {Cl}_{int}}{Q_{h}+{Cl}_{int}}$$

(4) $$HER=\frac{{Cl}_{hep}}{Q_{h}}$$

Here, A means the liver weight parameters and has values of 25.7, 32, 30, 87.5, and 40; B means the cell number parameters and has values of 139, 215, 120, 135, and 117; *Q_h_* represents hepatic blood flow and has values of 20.7, 30.9, 43.6, 90, and 55.2 mL/min/kg for humans, dogs, monkeys, mice, and rats, respectively [26]. Hepatic extraction ratios (HERs) of ≤0.25, 0.25–0.75, and ≥0.75 were regarded as low, moderate, and high, respectively [25].

The concentrations of the metabolites were quantitated, and their formation rates (pmol/mg protein/min) were calculated by dividing the amounts of metabolites formed from the incubation with human liver microsomes and human cDNA-expressed CYP or UGT enzymes by the incubation time. For the enzyme kinetic analysis, the formation rate of metabolites (pmol/mg protein/min) was plotted against the concentrations of JP-1366, and kinetic parameters such as *K*_m_, *V*_max_, Hill’s coefficient, and intrinsic clearance were calculated using enzyme kinetics software (SigmaPlot, version 12.5).

For the permeability calculation, the transport rate of JP-1366 and M1 was calculated from the slope of the regression line from the mean permeated amounts versus the incubation time plot. The permeability (P_app_) was calculated from the following equation [23,24]:

$$P_{app}(cm/s)=\frac{transport\;rate\left(nmol/s\right)}{JP-1366\;or\;M1\;concentration\;(\mu M)\times surface\;area\;of\;membrane\;({cm}^{2})}$$

Efflux ratio (ER) was calculated by dividing the P_app,BA_ value of JP-1366 or M1 by P_app,AB_. For the concentration dependency in ER of JP-1366 or M1, ERs of JP-1366 or M1 were plotted against the concentrations of JP-1366 or M1, and the inhibitory coefficient (IC_50_) was calculated using the inhibitory Emax model with SigmaPlot software (version 12).

Statistical significance for pharmacokinetic parameters and treatment groups was determined using Student’s *t*-test or one-way ANOVA accordingly. The values were treated as statistically significant when the *p*-value < 0.05.

## 3. Results

### 3.1. Metabolic Stability of JP-1366 in Hepatocytes

**Figure 1:**
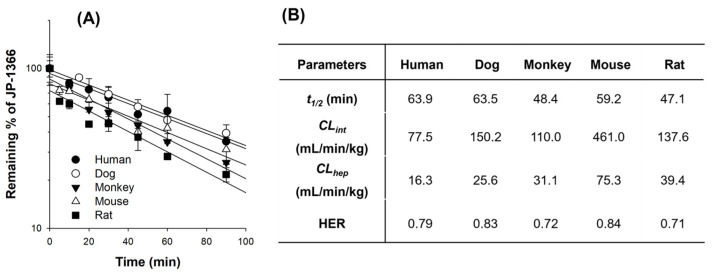
(**A**) Remaining percentages of JP-1366 after incubations of 1 µM JP-1366 with the hepatocytes of humans, dogs, monkeys, mice, and rats at 37 °C. Each datapoint represents mean ± SD (*n* = 3). (**B**) Elimination parameters from JP-1366 metabolic stability in human, dog, monkey, mouse, and rat hepatocytes. Parameters were calculated from the mean value of the remaining percentage of JP-1366 based on Equations (1)–(4) in Section 2.10.

The percentages of JP-1366 remained after the incubation of 1 µM of JP-1366 with the hepatocytes of humans, dogs, monkeys, mice, or rats at 37 °C are shown in Figure 1A. JP-1366 was gradually decreased in accordance with the incubation time, and the elimination half-life (t_1/2_) determined from the slope of Figure 1A was comparable among different species with a slightly higher elimination rate in rats and monkeys (Figure 1B). Figure 1B shows the metabolic parameters such as in vitro intrinsic clearance (*CL_int_*), hepatic clearance (*CL_hep_*), and HER in human, dog, monkey, mouse, or rat hepatocytes that were calculated using their physiological parameters such as liver weight, hepatic blood flow, etc. HER values of JP-1366 obtained from human, human, dog, monkey, mouse, or rat hepatocyte incubations were 0.79, 0.83, 0.72, 0.84, and 0.71, respectively, indicating that JP-1366 was highly metabolized in human, dog, and mouse hepatocytes but moderately metabolized in rat and monkey hepatocytes based on the decision criteria from the study of Bohnert et al. [25], suggesting the extensive hepatic metabolism of JP-1366. Therefore, the in vitro metabolisms of JP-1366 in human hepatocytes and human liver microsomes were further investigated.

### 3.2. In Vitro Metabolic Profiling of JP-1366 in Hepatocytes

**Figure 2:**
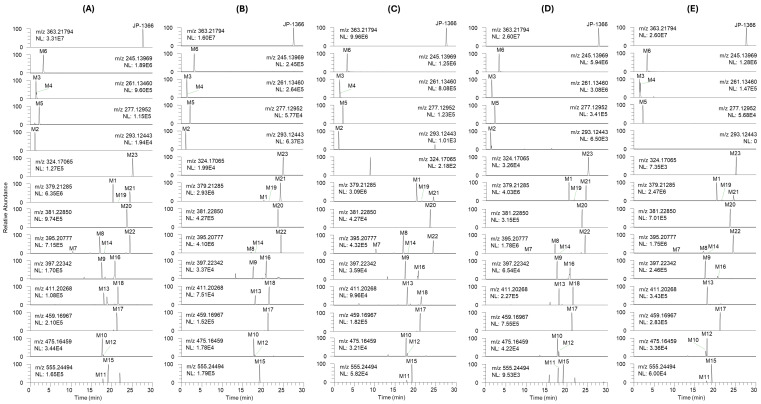
Representative extracted ion chromatograms of JP-1366 and its possible metabolites identified in (**A**) human, (**B**) dog, (**C**) monkey, (**D**) mouse, and (**E**) rat hepatocytes after incubation with 10 µM of JP-1366 for 60 min at 37 °C in CO_2_ incubator.

Using LC-HRMS analysis, the incubation of JP-1366 with human, dog, monkey, mouse, and rat hepatocytes resulted in the formation of 18 phase I metabolites and 5 phase II metabolites. Figure 2 shows the representative extracted ion chromatograms of JP-1366 and possible metabolites. Peaks for 23 possible metabolites were mostly detected in 5 hepatocytes and similar peak patterns regardless of hepatocytes from different species. Table 2 summarizes the retention time, the accurate mass of protonated molecular ions ([M+H]^+^), and product ions for possible 23 metabolites. All 23 metabolites were found in human hepatocytes, but M4 was not found in mouse hepatocytes. M2 and M18 were not found in rat hepatocytes. M7 and M11 were not found in dog hepatocytes and M23 was not found in monkey hepatocytes (Table 2). A total of 17 metabolites including M1, M3, M5, M6, M8, M9, M10, M12–M17, M19, and M20–M22 were determined after incubation with all 5 hepatocyte species (Table 2).

**Figure 3:**
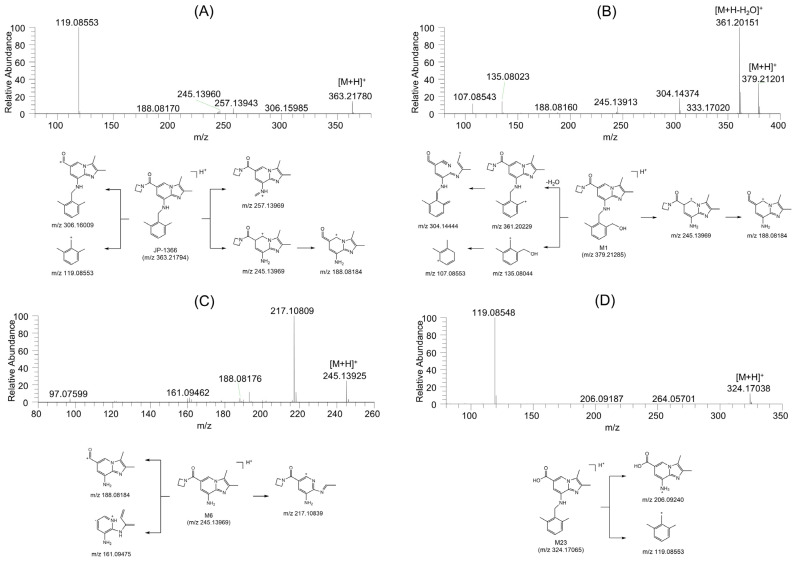
MS/MS spectra and possible fragmentation pattern of (**A**) JP-1366, (**B**) M1, (**C**) M6, and (**D**) M23.

We examined the fragmentation pattern of JP-1366 and potential metabolites in order to clarify the biotransformation pathway and compared it to that of available authentic standards. Three metabolites of JP-1366 such as M1, M6, and M23 were identified by comparison with the retention time and MS/MS spectra of the authentic standards (Figure 3). MS/MS spectra and possible fragmentation patterns of other possible 20 metabolites are shown in Supplementary Figures S1–S4. M2, M3, M4, and M5 were also formed from authentic standard M6 after incubation with human liver microsomes and NADPH. M3, M6, M7, M8, M9, M13, and M15 were also formed from authentic standard M1 in human liver microsomes with NADPH and UDPGA.

JP-1366 showed [M+H]^+^ ion at *m*/*z* 363.21820 and produced product ions at *m*/*z* 306.16034 (loss of the azetidinyl group from [M+H]^+^ ion), *m*/*z* 257,13969 (loss of the 2,6-dimethylphenyl group from [M+H]^+^ ion), *m*/*z* 245.13969 (loss of the 2,6-dimethylbenzyl group from [M+H]^+^ ion), and *m*/*z* 119.08553 ((2,6-dimethylphenyl)methylium ion) (Figure 3A).

M1 showed [M+H]^+^ ion at *m*/*z* 379.21285, which is 16 atomic mass units (amu) more than [M+H]^+^ ion of JP-1366, which suggested the hydroxylation of JP-1366. MS/MS spectrum of M1 showed product ions at *m*/*z* 361.20229 (loss of H_2_O from [M+H]^+^ ion), *m*/*z* 304.14444 (loss of the azetidinyl group from *m*/*z* 361.20229 ion), *m*/*z* 245.13969 (loss of the 2-(hydroxymethyl)-6-methylbenzyl group from [M+H]^+^ ion), and *m*/*z* 135.08044 ((2-(hydroxymethyl)-6-methylphenyl)methylium ion) (Figure 3B). M1 was identified as hydroxy-JP-1366 based on the retention time and MS/MS spectrum of the authentic standard.

M6 showed [M+H]^+^ ion at *m*/*z* 245.13969, which was 118 amu lower than the [M+H]^+^ ion of JP-1366, which indicated a loss of the 2,6-dimethylbenzyl group from the JP-1366. MS/MS spectrum of M6 and showed the product ions at *m*/*z* 217.10839 (loss of C_2_H_4_ at imidazole moiety from [M+H]^+^ ion) and *m*/*z* 188.08184 (loss of the azetidinyl group from [M+H]^+^ ion) (Figure 3C). M6 was identified as (8-amino-2,3-dimethylimidazo[1,2-a]pyridin-6-yl)(azetidin-1-yl)methanone based on the retention time and MS/MS spectrum of the authentic standard.

M23 showed [M+H]^+^ ion at *m*/*z* 324.17065, which was 39 amu less than the [M+H]^+^ ion of JP-1366, indicating the hydrolysis of JP-1366. The MS/MS spectrum of M24 showed the product ions at *m*/*z* 206.09240 (loss of the 2,6-dimethyl-benzyl group from [M+H]^+^ ion) and *m*/*z* 119.08553 (Figure 3D). M23 was identified as 8-((2,6-dimethylbenzyl)amino)-2,3-dimethylimidazo[1,2-a]pyridine-6-carboxylic acid, based on the retention time and MS/MS spectrum of the authentic standard.

M2 showed [M+H]^+^ ion at *m*/*z* 293.12443, which was 48 amu greater than the [M+H]^+^ ion of M6, indicating the trihydroxylation of M6. The MS/MS spectrum of M2 showed the product ions at *m*/*z* 275.11387 (loss of H_2_O from [M+H]^+^ ion), *m*/*z* 247.11895 (loss of CO from *m*/*z* 275.11387 ion), *m*/*z* 263.11387 (loss of CH_2_O at imidazole moiety from [M+H]+ ion), *m*/*z* 233.10330 (loss of CH_2_O from *m*/*z* 263.11387), *m*/*z* 221.10330, and *m*/*z* 203.09274 (Figure S1). M2 was presumed to be trihydroxy-M6, but the accurate positions of hydroxylation were not assigned devoid of the authentic standard.

M3 and M4 showed [M+H]^+^ ion at *m*/*z* 261.13460, which was 16 amu more than the [M+H]^+^ ion of M6, suggesting the monohydroxylation of M6. The MS/MS spectra of M3 and M4 showed the product ions at *m*/*z* 243.12404 (loss of H_2_O from [M+H]^+^ ion), *m*/*z* 233.10330 (loss of C_2_H_4_ from [M+H]^+^ ion), *m*/*z* 215.09274 (loss of C_2_H_4_ and H_2_O from [M+H]^+^ ion), and *m*/*z* 203.09274 (Figure S1). M3 and M4 were presumed to be hydroxy-M6, but the accurate position of hydroxylation was not assigned devoid of the authentic standard.

M5 showed [M+H]^+^ ion at *m*/*z* 277.12952, which was 32 amu more than the [M+H]^+^ ion of M6, suggesting the dihydroxylation of M6. The MS/MS spectrum of M5 showed the product ions at *m*/*z* 217.10839 (2-((3-amino-5-(azetidine-1-carbonyl)pyridin-2(1H)-ylidene)amino) ethen-1-ylium ion), *m*/*z* 205.10839 (3-amino-5-(azetidine-1-carbonyl)-N-methylenepyridin-2(1H)-iminium ion), and *m*/*z* 193.08458 (Figure S1), indicating that M5 might be dihydroxy-M6 at the methyl group of imidazole moiety.

M7 showed [M+H]^+^ ion at *m*/*z* 395.20777, which was 16 amu more than the [M+H]^+^ ion of M1, indicating the hydroxylation of M1. The MS/MS spectrum of M7 showed the product ions at *m*/*z* 377.19720 (loss of H_2_O from [M+H]^+^ ion), *m*/*z* 359.18664 (loss of H_2_O from *m*/*z* 377.19720 ion), *m*/*z* 245.13969, and *m*/*z* 151.07536 ((hydroxy-2-(hydroxymethyl)-6-methylphenyl)methylium) (Figure S2), indicating that M7 might be hydroxy-M1, but the accurate position of hydroxylation at methylphenyl moiety was not assigned devoid of the authentic standard.

M8 showed [M+H]^+^ ion at *m*/*z* 395.20777, which was 16 amu more than the [M+H]^+^ ion of M1, indicating the hydroxylation of M1. The MS/MS spectrum of M8 showed the product ions at *m*/*z* 377.19720 (loss of H_2_O from [M+H]^+^ ion), *m*/*z* 320.13935 (loss of the azetidinyl group from *m*/*z* 377.19720 ion), *m*/*z* 261.13460 (loss of the 2-(hydroxymethyl)-6-methylbenzyl group from [M+H]^+^ ion), *m*/*z* 204.07675 (loss of the azetidinyl group from *m*/*z* 261.13460 ion), and *m*/*z* 135.08044, indicating that M8 might be hydroxy-M1 (Figure S2), but the accurate position of hydroxylation at 2,3-dimethylimidazo[1,2-a]pyridine moiety was not assigned devoid of the authentic standard.

M9 showed [M+H]^+^ ion at *m*/*z* 397.22342, which was 18 amu more than the [M+H]^+^ ion of M1, indicating the hydroxylation and reduction of M1. The MS/MS spectrum of M9 showed the product ions at *m*/*z* 379.21285 (loss of H_2_O from [M+H]^+^ ion), *m*/*z* 322.15500 (loss of the azetidinyl group from *m*/*z* 379.21285 ion), *m*/*z* 304.14444 (loss of H_2_O from *m*/*z* 322.15500 ion), *m*/*z* 263.15025 (loss of the 2-(hydroxymethyl)-6-methylbenzyl group from [M+H]^+^ ion), and *m*/*z* 135.08044, indicating that M9 might be reduced hydroxy-M1 (Figure S2), but the accurate positions of hydroxylation and reduction at (2,3-dimethylimidazo[1,2-a]pyridin-6-yl)methanone moiety were not assigned devoid of the authentic standard.

M13 showed [M+H]^+^ ion at *m*/*z* 411.20268, which was 32 amu more than the [M+H]^+^ ion of M1, indicating the dihydroxylation of M1. The MS/MS spectrum of M13 showed the product ions at *m*/*z* 393.19212 (loss of H_2_O from [M+H]^+^ ion), *m*/*z* 333.17099 (loss of the two CH_2_OH groups and rearrangement from *m*/*z* 393.19212 ion), *m*/*z* 277.12952 (loss of the 2-(hydroxymethyl)-6-methylbenzyl group from [M+H]^+^ ion), and *m*/*z* 135.08044 (Figure S2). Moreover, M13 was formed from authentic standard M1 incubated with human liver microsomes and NADPH at 37 °C, suggesting that M13 may be dihydroxy-M1 formed by the subsequent dihydroxylation of M1.

M15 showed [M+H]^+^ ion at *m*/*z* 555.24494, which is 176 amu more than the [M+H]^+^ ion of M1, and produced similar product ions compared to M1, suggesting that the glucuronidation occurs at the hydroxyl group of M1. The MS/MS spectrum of M15 showed product ions at *m*/*z* 379.21285 (loss of the glucuronyl group from [M+H]^+^ ion), *m*/*z* 361.20229 (loss of H_2_O from *m*/*z* 379.21285 ion), *m*/*z* 304.14444, *m*/*z* 245.13969, and *m*/*z* 135.08044 (Figure S2), indicating that M15 might be M1 glucuronide. Moreover, M15 was formed from M1 after incubation with human liver microsomes and UDPGA at 37 °C.

M19 showed [M+H]^+^ ion at *m*/*z* 379.21285, which is 16 amu more than the [M+H]^+^ ion of JP-1366, indicating the hydroxylation of JP-1366. The MS/MS spectrum of M19 showed the product ions at *m*/*z* 322.15500 (loss of the azetidinyl group from [M+H]^+^ ion), *m*/*z* 257.13969, *m*/*z* 245.13969, and *m*/*z* 135.08044 ((hydroxy-2,6-dimethylphenyl)methylium ion) (Figure S3), indicating that M19 might be hydroxy-JP-1366. The accurate position of hydroxylation at phenyl moiety was not assigned devoid of the authentic standard.

M11 showed [M+H]^+^ ion at m/z 555.24494, which was 192 amu more than the [M+H]^+^ ion of JP-1366, indicating the hydroxylation and glucuronidation of JP-1366. The MS/MS spectrum of M11 showed the product ions at *m*/*z* 379.21285 (loss of the glucuronyl group from [M+H]^+^ ion), *m*/*z* 311.11253 (loss of *m*/*z* 245.13969 from [M+H]^+^ ion), *m*/*z* 257.13969, *m*/*z* 245.13969, and *m*/*z* 135.08044 ((hydroxy-2,6-dimethylphenyl)methylium) (Figure S3). M11 was not formed from M1 after incubation with human liver microsomes and UDPGA at 37 °C, and the β-glucuronidase treatment of JP-1366-treated hepatocytes resulted in both the increase in M19 and the decrease in M11. These results support that M11 may be hydroxy-JP-1366, an M19 glucuronide.

M17 showed [M+H]^+^ ion at *m*/*z* 459.16967, which was 96 amu more than the [M+H]^+^ ion of JP-1366, indicating the hydroxylation and sulfation of JP-1366. The MS/MS spectrum of M17 showed the product ions at *m*/*z* 379.21285 (loss of the SO_3_ group from [M+H]^+^ ion), *m*/*z* 322.15500 (loss of the azetinyl group from *m*/*z* 379.21285 ion), *m*/*z* 257.13969, *m*/*z* 245.13969, *m*/*z* 215.03726 ((2,6-dimethyl-sulfooxy-phenyl)methylium ion), and *m*/*z* 135.08044 (Figure S3). M17 was not formed from M1 incubated with human liver S9 fraction and PAPS at 37 °C, and the sulfatase treatment of JP-1366-treated hepatocytes resulted in both the increase in M19 and the decrease in M17. These results indicated that M17 might be hydroxy-JP-1366, an M19 sulfate.

M14 showed [M+H]^+^ ion at *m*/*z* 395.20777, which was 32 amu more than the [M+H]^+^ ion of JP-1366, indicating the dihydroxylation of JP-1366. The MS/MS spectrum of M14 showed the product ions at *m*/*z* 338.14992 (loss of the azetidinyl group from [M+H]^+^ ion), *m*/*z* 261.13460 (loss of the hydroxy-2,6-dimethyl-benzyl group from [M+H]^+^ ion), *m*/*z* 204.07675 (loss of the azetidinyl group from *m*/*z* 261.13460 ion), and *m*/*z* 135.08044 ((hydorxy-2,6-dimethylphenyl)methylium ion) (Figure S3). M14 was not formed from M1 incubated with human liver microsomes and NADPH at 37 °C. These results support that M14 may be dihydroxy-JP-1366 via the hydroxylation of M19, but the accurate position of hydroxylation was not assigned devoid of the authentic standard.

M10 and M12 showed [M+H]^+^ ion at *m*/*z* 475.16459, which was 112 amu more than the [M+H]^+^ ion of JP-1366, indicating the dihydroxylation and sulfation of JP-1366. The MS/MS spectra of M10 and M12 showed the product ions at *m*/*z* 395.20777 (loss of the SO_3_ group from [M+H]^+^ ion), *m*/*z* 377.19720 (loss of H_2_O from *m*/*z* 395.20777 ion), *m*/*z* 338.14992 (loss of the azetidinyl group from *m*/*z* 395.20777 ion), *m*/*z* 261.13460, *m*/*z* 204.07675, and *m*/*z* 135.08044 (Figure S3). M10 and M12 were not identified from M1 incubated with human liver S9 fraction, PAPS, and NADPH at 37 °C, and the sulfatase treatment of JP-1366-treated hepatocytes resulted in both the increase in M14 and the decrease in M10 and M12. These results indicate that M10 and M12 might be dihydroxy-JP-1366 sulfate formed via the sulfation of M19, but the accurate positions of hydroxylation and sulfation were not assigned devoid of the authentic standard.

M21 showed [M+H]^+^ ion at *m*/*z* 379.21285, which was 16 amu more than the [M+H]^+^ ion of JP-1366, indicating the hydroxylation of JP-1366. The MS/MS spectrum of M21 showed the product ions at *m*/*z* 349.20229 (loss of CH_2_O from [M+H]^+^ ion), *m*/*z* 273.13460 (loss of the 2,6-dimethylphenyl group from [M+H]^+^ ion), *m*/*z* 243.12355 (loss of CH_2_O from *m*/*z* 273.13400 ion), and *m*/*z* 119.08553 (Figure S4), suggesting that M21 might be hydroxy-JP-1366 via hydroxylation at 2,3-dimethylimidazole moiety.

M16 showed [M+H]^+^ ion at *m*/*z* 397.22342, which was 34 amu more than the [M+H]^+^ ion of JP-1366, indicating the dihydroxylation of and reduction in JP-1366. The MS/MS spectrum of M16 showed the product ions at *m*/*z* 367.21285 (loss of CH_2_O from [M+H]^+^ ion), *m*/*z* 291.14517 (loss of the 2,6-dimethylphenyl group from [M+H]^+^ ion), *m*/*z* 261.13460 (loss of the 2,6-dimethylphenyl group and CH_2_OH from [M+H]^+^ ion), and *m*/*z* 119.08553 (Figure S4), suggesting that M16 might be reduced dihydroxy-JP-1366 via dihydroxylation and reduction at (2,3-dimethylimidazo[1,2-a]pyridin-6-yl)methanone moiety.

M20 showed [M+H]^+^ ion at *m*/*z* 381.22850, which was 18 amu more than the [M+H]^+^ ion of JP-1366, indicating the hydroxylation of and reduction in JP-1366. The MS/MS spectrum of M21 showed the product ions at *m*/*z* 306.16009 (loss of H_2_O and azetidinyl moiety from [M+H]^+^ ion), *m*/*z* 275.15025 (loss of the 2,6-dimethylphenyl group from [M+H]^+^ ion), and *m*/*z* 119.08553 (Figure S4), suggesting that M20 might be reduced hydroxy-JP-1366. The accurate position of hydroxylation at 2,3-dimethylimidazole moiety was not assigned devoid of the authentic standard.

M22 showed [M+H]^+^ ion at *m*/*z* 395.20777, which was 32 amu more than the [M+H]^+^ ion of JP-1366, indicating the dihydroxylation of JP-1366. The MS/MS spectrum of M22 showed the product ions at *m*/*z* 289.12952 (loss of the 2,6-dimethylphenyl group from [M+H]^+^ ion), *m*/*z* 277.12952 (loss of the 2,6-dimethylbenzyl group from [M+H]^+^ ion), *m*/*z* 188.08184, and *m*/*z* 119.08553 (Figure S4), suggesting that M22 might be dihydroxy-JP-1366. The accurate position of dihydroxylation at 2,3-dimethylimidazole moiety was not assigned devoid of the authentic standard.

M18 showed [M+H]^+^ ion at *m*/*z* 411.20268, which was 48 amu more than the [M+H]^+^ ion of JP-1366, indicating the trihydroxylation of JP-1366. The MS/MS spectrum of M18 showed the product ions at *m*/*z* 381.19212 (loss of CH_2_O from [M+H]^+^ ion), *m*/*z* 305.12443 (loss of the 2,6-dimethylphenyl group from [M+H]^+^ ion), *m*/*z* 275.11387 (loss of the 2,6-dimethylphenyl group and CH_2_O from [M+H]^+^ ion), and *m*/*z* 119.08546 (Figure S4), suggesting that M18 might be trihydroxy-JP-1366. The accurate position of trihydroxylation at 2,3-dimethylimidazopyridine moiety was not assigned devoid of the authentic standard.

Collectively, *N*-dearylation, hydroxylation, dihydroxylation, trihydroxylation, hydrogenation, hydrolysis, glucuronidation, sulfation, and their combination were major in vitro metabolic pathways involved in the metabolism of JP-1366, and most metabolites were found in the hepatocytes of humans, dogs, monkeys, mice, and rats.

### 3.3. Characterization of CYP Isozymes Involved in JP-1366 Metabolism

**Figure 4:**
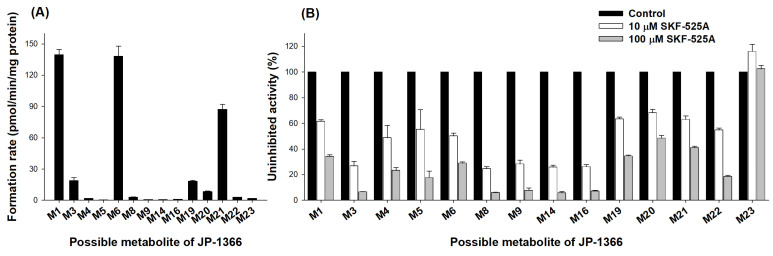
(**A**) Formation rate of possible JP-1366 metabolites after the incubation of 5 µM of JP-1366 with human liver microsomes in the presence of NADPH. (**B**) Inhibitory effect of SKF-525A (10 μM and 100 μM; a nonselective CYP inhibitor) on JP-1366 metabolism in human liver microsomes.

To screen the involvement of CYP isozymes, we assessed the inhibitory effect of SKF-525A, a nonselective CYP inhibitor [27], on JP-1366 metabolism in human liver microsomes (Figure 4). Among the 23 metabolites, 4 phase I metabolites such as M2, M7, M13, and M18 and phase II metabolites (M10, M11, M12, M15, and M17) were not detected from the incubation of 5 μM of JP-1366 in human liver microsomes with NADPH. M1, M6, and M21 seemed to be the primary metabolites of JP-1366 in human liver microsomes, which was consistent with the results from human hepatocytes (Figure 4A). SKF-525A inhibited the formation of 13 phase I metabolites such as M1, M3, M4, M5, M6, M8, M9, M14, M16, M19, M20, M21, and M22, which supported that CYP enzymes may play the major roles in the metabolism of JP-1366 in a concentration-dependent manner (Figure 4B). However, the formation of M23 from JP-1366 was not inhibited by SKF-525A treatment, but the treatment of NaF, a nonselective esterase inhibitor, inhibited the formation of M23 (18.9% ± 3.9% inhibition at 100 μM NaF, 73.3% ± 0.3% inhibition at 1000 μM NaF). These findings showed that M23 was formed from JP-1366 by esterase.

**Figure 5:**
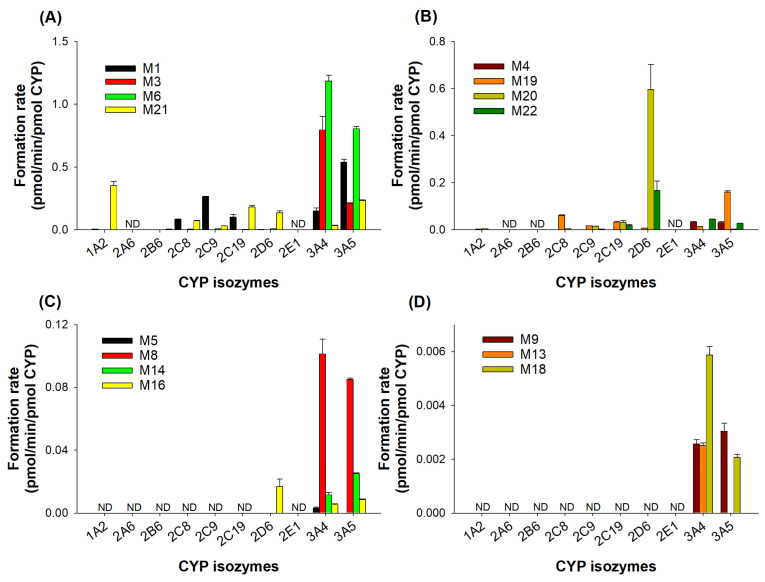
Formation rates of possible JP-1366 metabolites formed after the incubation of 5 µM of JP-1366 with human cDNA-expressed CYP isozymes (CYP1A2, 2A6, 2B6, 2C8, 2C9, 2C19, 2D6, 2E1, 3A4, and 3A5) in the presence of NADPH at 37 °C for 30 min. (**A**) M1, M3, M6, and M21; (**B**) M4, M19, M20, and M22; (**C**) M5, M8, M14, and M16; (**D**) M9, M13, and M18. ND: not detected. Each datapoint represents mean ± SD (*n* = 3).

To elucidate the responsible CYP isozyme for JP-1366 metabolism, the formation rates of phase I metabolites formed from 5 μM of JP-1366 were determined by incubating with 10 human cDNA-expressed CYPs (1A2, 2A6, 2B6, 2C8, 2C9, 2C19, 2D6, 2E1, 3A4, and 3A5). JP-1366 metabolism involved several CYP isozymes such as CYP1A2, 2C8, 2C9, 2C19, 2D6, 3A4, and 3A5 (Figure 5). Among them, CYP3A4 and CYP3A5 played major roles in the metabolism of JP-1366 to M1, M3, M4, M5, M6, M8, M9, M13, M14, M16, M18, and M19. CYP1A2, 2C8, 2C9, 2C19, 3A4, and 3A5 played the roles in M21 formation (Figure 5A). CYP2D6 is mainly involved in the formation of M20 and M22 (Figure 5B).

**Figure 6:**
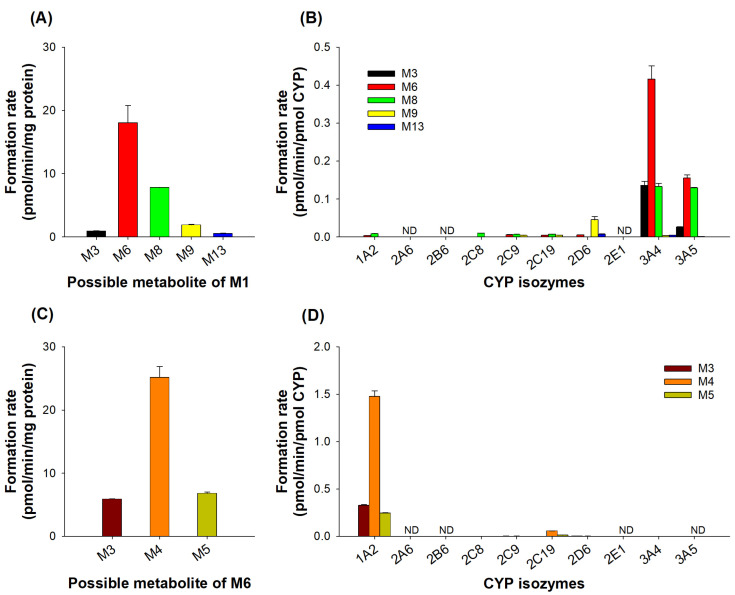
(**A**) The formation rate of subsequent metabolites after the incubation of 5 µM of M1 with human liver microsomes in the presence of NADPH at 37 °C for 30 min. (**B**) The formation rate of M3, M6, M8, M9, and M13 after the incubation of 5 µM of M1 with human cDNA-expressed CYP isozymes (1A2, 2A6, 2B6, 2C8, 2C9, 2C19, 2D6, 2E1, 3A4, and 3A5) in the presence of NADPH at 37 °C for 30 min. (**C**) The formation rate of subsequent metabolites after the incubation of 5 µM of M6 with human liver microsomes. (**D**) The formation rate of M3, M4, and M5 after the incubation of 5 µM of M6 with human cDNA-expressed CYP isozymes. ND: not detected. Each datapoint represents a mean ± SD (*n* = 3).

We examined subsequent metabolites after the incubation of M1 and M6 with human liver microsomes and human cDNA-expressed CYP isozymes in the presence of NADPH in order to determine the subsequent metabolism of JP-1366 metabolites. At first, following the incubation of M1 with human liver microsomes, M3, M6, M8, M9, and M13 were formed from M1. Among these metabolites, M6 showed the highest formation rate (Figure 6A). In order to identify the CYP isozymes involved in these subsequent metabolisms, the formation rates of M3, M6, M8, M9, and M13 metabolites from M1 were evaluated by incubating with 10 human cDNA-expressed CYPs (1A2, 2A6, 2B6, 2C8, 2C9, 2C19, 2D6, 2E1, 3A4, and 3A5). The incubation of M1 with CYP3A4 and 3A5 isozymes transformed into M3, M6, and M8, and CYP2D6 was involved in the transformation from M1 to M9 (Figure 6B). In the case of M6 incubation with human liver microsomes, M3, M4, and M5 were formed, with M4 having the highest formation rate (Figure 6C). CYP1A2 played a major role in the progression of metabolism from M6 to the formation of M3, M4, and M5 (Figure 6D).

**Figure 7:**
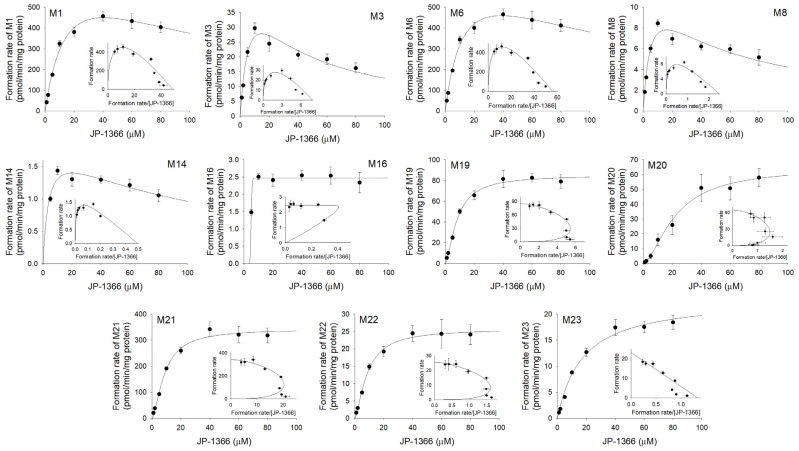
Concentration-dependent formation rates of JP-1366 metabolites in human liver microsomes in a concentration range of 1–80 μM of JP-1366. Insets are Eadie–Hofstee plots. Each datapoint represents mean ± SD (*n* = 3).

Enzyme kinetic profiles for the formation of hydroxy-JP-1366 (M1, M19, and M21), dihydroxy-JP-1366 (M8, M14, and M22) and M3, M6, and M20 from JP-1366 in human liver microsomes and cDNA-expressed CYP1A2, 2C8, 2C9, 2C19, 2D6, 3A4, and 3A5 isozymes in the presence of NADPH were fitted to substrate inhibition kinetics or the Hill equation (Figure 7, Supplementary Figure S5). The enzyme kinetic parameters are summarized in Table 3. The formation of M1, M3, M6, M8, and M14 in human liver microsomes showed substrate inhibition kinetics, and the formation of M16, M19, M20, M21, and M22 could be analyzed using the Hill equation. The formation of M23 from JP-1366 in human liver microsomes showed simple Michaelis–Menten kinetics (Figure 7). However, the concentration-dependent formations of M4, M5, and M9 from JP-1366 in human liver microsomes were not observed.

The order of the formation of major phase I metabolites from JP-1366 in human liver microsomes is as follows: M6 (CL_int_: 55.9 µL/min/mg protein) > M1 (CL_int_: 48.6 µL/min/mg protein) > M21 (CL_int_: 38.9 µL/min/mg protein) > M19 (CL_int_: 10.3 µL/min/mg protein) > M3 (CL_int_: 7.8 µL/min/mg protein) > M22 (CL_int_: 3.0 µL/min/mg protein) > M20 (CL_int_: 2.9 µL/min/mg protein) > M8 (CL_int_: 2.5 µL/min/mg protein) > M23 (CL_int_: 1.3 µL/min/mg protein) > M14 (CL_int_: 0.5 µL/min/mg protein) (Table 3). This indicates that the *N*-dearylation of JP-1366 to M6 and the hydroxylation of JP-1366 to M1, M19, and M21 might be major metabolic pathways.

Then, we analyzed the enzyme kinetic parameters for CYP isozymes that were involved in the formation of JP1366 metabolites and expressed their relative contribution (Table 3). CYP3A4 and CYP3A5 isozymes played major roles in the formation of hydroxy-JP-1366 (M1, M19, and M21); dihydroxy-JP-1366 (M8, M14, and M22); and M3, M6, and M16 from JP-1366 (Supplementary Figure S5, Table 3). CYP1A2 played a role in the formation of hydroxy-JP-1366 (M21) and M20 from JP-1366. CYP2C8 played a minor role in the formation of hydroxy-JP-1366 (M1, M19, and M21). CYP2C9 played a minor role in the formation of hydroxy-JP-1366 (M1, M19, and M21) and M6 from JP-1366. CYP2C19 played a minor role in the formation of hydroxy-JP-1366 (M1, M19, and M21) and M6, M20, and M22 from JP-1366. CYP2D6 played a major role in the formation of M21 but a minor role in the formation of M6, M16, M19, and M22 from JP-1366 (Table 3).

**Figure 8:**
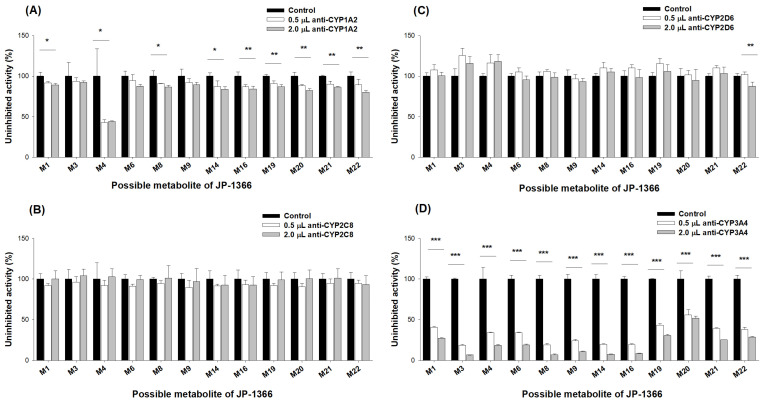
Effects of human-specific CYP antibodies on the formation rate of metabolites formed from 5 µM of JP-1366. Ultrapooled human liver microsomes (0.2 mg protein/mL) were preincubated with (**A**) anti-CYP1A2, (**B**) anti-CYP2C8, (**C**) anti-CYP2D6, and (**D**) anti-CYP3A4. Each datapoint represents mean ± SD (*n* = 3). *: *p* < 0.05; **: *p* < 0.01; ***: *p* < 0.001 significantly different from one-way ANOVA test.

We investigated the inhibitory effects of CYP1A2, 2C8, 2D6, and 3A4 antibodies on JP-1366 metabolism in human liver microsomes (HLMs) to further clarify the major metabolic enzymes among the multiple enzymes that are involved in the phase I metabolism of JP-1366. The pretreatment of anti-CYP1A2 substantially decreased the formation rate of M4 (Figure 8A), which was consistent with the major involvement of CYP1A2 in the formation of M4 from M6 (Figure 6D). Even though the CYP1A2 antibody significantly inhibited the formation of M1, M8, M14, M16, M19, M21, and M22, it is decreased by a small change. It suggested the minor involvement of CYP1A2 in the metabolism of JP-1366 (Figure 8A). Neither the CYP2C8 antibody nor the CYP2D6 antibody profoundly decreased the formation of JP-1366 metabolites, which suggested the limited involvement of CYP2C8 and CYP2D6 in the formation of JP-1366 metabolites (Figure 8B,C). However, the pretreatment of the CYP3A4 antibody significantly reduced the formation of most JP-1366 metabolites (i.e., M1, M3, M4, M6, M8, M9, M14, M16, M19, M20, M21, and M22) in HLMs (Figure 8D).

### 3.4. Characterization of UGT Enzymes Responsible for M1 Glucuronidation

**Figure 9:**
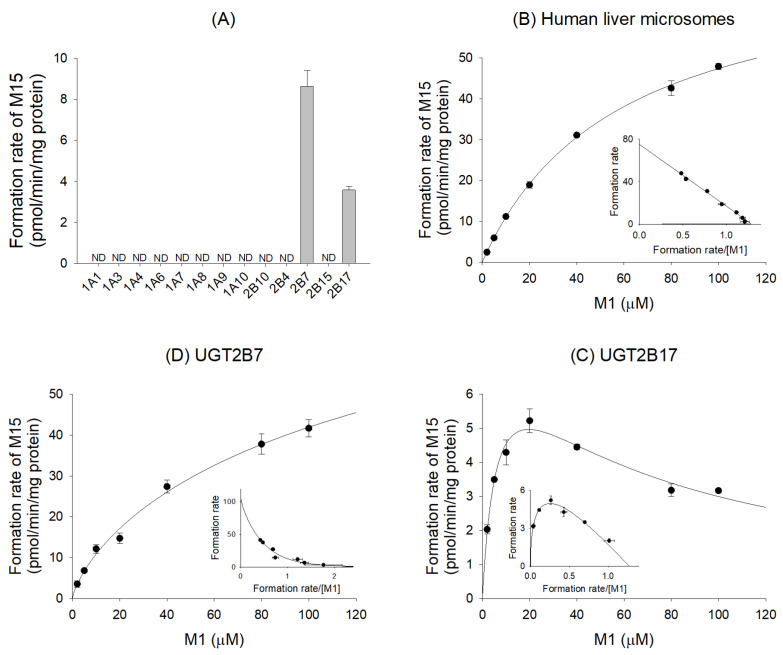
(**A**) Formation rate of M15 (JP-1366 M1 glucuronide) formed via the incubation of 10 µM of M1 with human cDNA-expressed UGT enzymes (1A1, 1A3, 1A4, 1A6, 1A7, 1A8, 1A9, 1A10, 2B4, 2B7, 2B10, 2B15, and 2B17) in the presence of UDPPGA. Concentration-dependent formation rates of M15 (JP-1366 M1 glucuronide) in the concentration range of 2–100 μM of M1 in (**B**) human liver microsomes and human cDNA-expressed (**C**) UGT2B7 and (**D**) UGT2B17 supersomes. Insets are Eadie–Hofstee plots. ND: not detected. Each datapoint represents mean ± SD (*n* = 3).

In order to characterize UGT enzymes responsible for the metabolism of M1 to M15 (M1 glucuronide), the formation rates of M15 from 10 μM of M1 were evaluated via incubation with 11 human cDNA-expressed UGTs (1A1, 1A3, 1A4, 1A6, 1A7, 1A8, 1A9, 1A10, 2B4, 2B7, 2B10, 2B15, and 2B17) and UDPGA at 37 °C for 30 min. UGT2B7 and UGT2B17 were responsible for the glucuronidation of M1 (Figure 9A). The enzyme kinetic profiles for the glucuronidation of M1 to M15 followed the Hill equation in HLMs (Figure 9B), which yielded kinetic parameters such as *K*_m_ of 57.9 μM, *V*_max_ of 74.9 pmol/min/mg protein, and *CL_int_* of 1.29 μL/min/mg protein (Table 4). Enzyme kinetics for the formation of M15 from M1 in human cDNA-expressed UGT2B7 and UGT2B17 enzymes represented the Hill equation and substrate inhibition mode, respectively (Figure 9C and Figure 9D). Kinetic parameters calculated from UGT2B7 were similar to those in HLMs and *CL_int_* for the formation of M15 in UGT2B17, which was higher than that of UGT2B7. Therefore, we can conclude that UGT2B17 rather than UGT2B7 is primarily responsible for the formation of M15.

### 3.5. Elucidation of Metabolic Pathway of JP-1366

**Figure 10:**
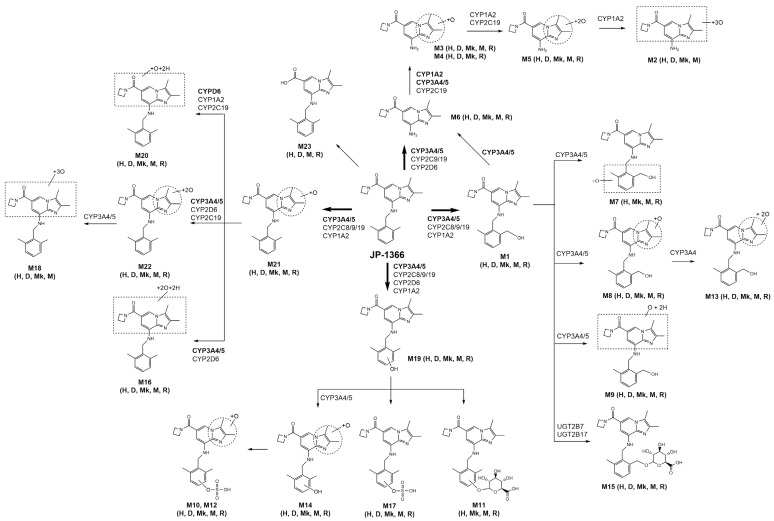
Metabolic pathways of JP-1366 in human (H), dog (D), monkey (Mk), mouse (M), and rat (R) hepatocytes and responsible metabolic enzymes.

Based on the identification of possible JP-1366 metabolites and associated CYP and UGT enzymes, we might suggest the in vitro metabolic pathways of JP-1366 in human, rat, mouse, dog, and monkey hepatocytes (Figure 10).

### 3.6. Screening of Substrate Specificity of JP-1366 and M1 on Drug Transporters

We investigated the uptake of JP-1366 and M1 in the presence or absence of typical transporter inhibitors in HEK293 cells expressing MATE1/2K, OCT1/2, OAT1/3, and OATP1B1/1B3 transporters. The substrate specificity for eight SLC transporters was determined when the fold increase of JP-1366 and M1 in HEK293 cells overexpressing SLC transporters compared to HEK293-mock cells was more than two and the inhibition was more than 50% in the fold increase through the addition of typical transporter inhibitors [13,15,28]. As shown in Table 4, the uptake of JP-1366 was not significantly increased in HEK293 cells expressing MATE1/2K, OCT1/2, OAT1/3, and OATP1B1/1B3 transporters compared with HEK293-mock cells. Furthermore, adding representative inhibitors did not significantly change the uptake of JP-1366. These results indicated that MATE1/2K, OCT1/2, OAT1/3, and OATP1B1/1B3 may not be involved in the uptake of JP-1366. The same results were observed for M1 (Table 5). It also suggested that MATE1/2K, OCT1/2, OAT1/3, and OATP1B1/1B3 may not be involved in the uptake of M1.

The involvement of P-gp and BCRP in the transport of JP-1366 and M1 was investigated in LLC-PK1 cells overexpressing P-gp and BCRP. Table 5 shows the apparent permeability (P_app_) and ER change of JP-1366 in LLC-PK1-P-gp and BCRP cells. P-gp or BCRP substrate specificity was determined when ER was more than two and the ER change was more than 50% through the addition of CsA or Ko134 [13,15,28]. The ER of JP-1366 was 1.3 and lower than 2.0, which indicated that JP-1366 seemed not to be a P-gp substrate. Moreover, the addition of 20 μM of CsA did not modulate the ER of JP-1366, supporting the idea that JP-1366 might not be a substrate for P-gp (Table 6). The ER of JP-1366 was 1.4 and lower than 2.0, which indicated that JP-1366 seemed to not be a BCRP substrate. Moreover, the addition of 1 μM of Ko134 did not modulate the ER of JP-1366, which supports the idea that JP-1366 might not be a substrate for BCRP (Table 6).

The ER of M1 was 14.2 and higher than 2.0, which indicated that JP-1366 M1 seemed to be a substrate for P-gp. The addition of 20 μM of CsA decreased the ER of M1 to 3.4, which supports the idea that M1 might be a substrate for P-gp (Table 6). The ER of M1 was 1.4 and lower than 2.0, which indicated that M1 seemed to not be a BCRP substrate. Additionally, the addition of 1 μM of Ko134 did not modulate the ER of M1 (Table 6).

**Figure 11:**
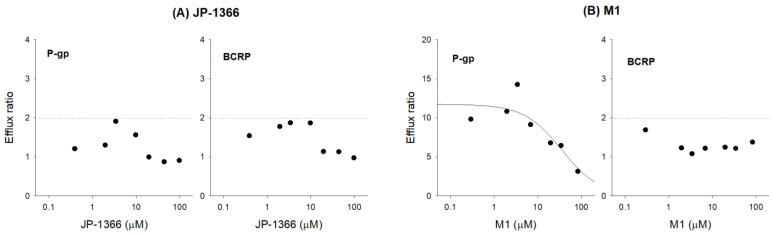
Concentration dependence in the efflux ratio of (**A**) JP-1366 (0.4–100 μM) and (**B**) M1 (0.3–85 μM) was measured in LLC-PK1-P-gp and LLC-PK1-BCRP cell monolayer. The bar represents the mean ± SD (*n* = 3) and the ER was calculated by dividing mean B to A P_app_ (P_app,BA_) by mean A to B P_app_ (P_app,AB_) value of JP-1366 or M1.

We examined the concentration dependency in the ER of JP-1366 and M1 in the concentration range of 0.3–100 μM in LLC-PK1-P-gp cells and in LLC-PK1-BCRP cells. The ER of JP-1366 in LLC-PK1-P-gp cells was 0.9–1.9 in the concentration range of 0.4–100 μM, which was lower than 2.0. When the findings of the substrate screening were taken together, we were able to confirm that JP-1366 might not be a substrate for P-gp in LLC-PK1-P-gp cells. The ER of JP-1366 in LLC-PK1-BCRP cells was 0.9–1.9 in the concentration range of 0.4–100 μM, which was lower than 2.0. Again, we confirmed that JP-1366 may not be a substrate for BCRP (Figure 11A).

The ER of M1 in LLC-PK1-P-gp cells was 14.2–3.2 in the concentration range of 0.3–85 μM and decreased in a concentration-dependent manner. The results suggested that JP-1366 M1 is a substrate for P-gp and the half-maximal saturation concentration (K_m_) for the ER of M1 was 35.8 μM in LLC-PK1-P-gp cells. The ER of M1 in LLC-PK1-BCRP cells was 1.1–1.7 in the concentration range of 0.3–85 μM, indicating that M1 may not be a substrate for BCRP in LLC-PK1-BCRP cells (Figure 11B).

### 3.7. Permeability of JP-1366 and M1 in Caco-2 Cells

Caffeine and propranolol were used as highly permeable markers. Ofloxacin and atenolol were used as moderate- and low-permeability marker compounds, respectively. The P_app_ of caffeine, propranolol, ofloxacin, and atenolol were similar to the previous reports and the efflux ratios of all marker compounds were close to 1.0 (Table 7) [20,29,30]. The results demonstrated that our Caco-2 system may be used to investigate the permeability of new molecular entities.

The absorptive permeability (A to B P_app_) of 4 μM of JP-1366 was similar to the permeability of caffeine, a marker compound for highly permeable markers (Table 7). The secretory permeability (B to A P_app_) of 4 μM of JP-1366 was 10.70 ± 1.77 × 10^−6^ cm/s and the ER was 0.47, which indicated the favorable absorption in the intestinal absorption of JP-1366 and is consistent with the limited contribution of efflux transporters such as P-gp and BCRP (Table 5 and Table 6). However, the A to B P_app_ of M1 was similar to the permeability of ofloxacin, a marker compound for moderately permeable markers (Table 7). The B to A P_app_ of JP-1366 was 20.30 ± 1.51 × 10^−6^ cm/s and the ER was 6.0, which suggested that efflux transporters in the intestinal lumen might act as a moderate permeability of M1 and is also consistent with the involvement of P-gp in the efflux of M1 (Table 6).

## 4. Discussion

Due to the several limitations of PPIs, such as 2–3 days of lag time to reach the steady state of acid inhibition [8,31] and large inter-individual variability and high drug–drug interaction potential by the CYP2C19-mediated metabolism of PPIs [32,33], the role of CYP2C19 in the metabolism of the P-CAB drug has been of great interest. In this regard, the clinically prescribed P-CAB drugs vonoprazan, tegoprazan, and fexuprazan have some advantages due to their higher stability, higher stomach distribution, and direct inhibition of H^+^/K^+^-ATPase, which have led to faster acid inhibition, better nighttime gastric acid suppression, less dependence on CYP2C19-mediated metabolism, and no food effect [11].

In this study, we focused on the identification of the potential metabolites of JP-1366 (zastaprazan) and elucidated the role of CYP and UGT isozymes in the formation of major metabolites of this compound in order to understand the predictive pharmacokinetics and drug interactions. JP-1366 undergoes extensive HER and produces 23 metabolites (18 phase I metabolites and 5 phase II metabolites) in human hepatocytes. It also showed high HER values in the range of 0.71–0.84 in dog, monkey, mouse, and rat hepatocytes (Figure 1). Although the in vivo metabolites of JP-1366 need to be investigated, the results suggested the increased contribution of metabolism as a pharmacokinetic determinant of JP-1366. From the enzyme kinetic studies of JP-1366 using HLMs, M1, M19, M21 (hydroxy-JP-1366 metabolites), and M6 (*N*-dearylated metabolite of JP-1366) seemed to be the major metabolites of this compound (Figure 7). That is, JP-1366 was also mainly metabolized to M6 via CYP3A4/5 (97.0%) with little contribution from CYP2C9, CYP2C19, and CYP2D6, and hydroxy-JP-1366 metabolites (M1, M19, and M21) via CYP3A4/5 (85.9%, 86.5%, and 82.7%, respectively) with minor contribution from CYP1A2, CYP2C8, CYP2C9, CYP2C19, and CYP2D6 (Table 3, Figure 10). Anti-CYP3A antibody treatment significantly decreased the formation of the major metabolites such as M1, M6, M19, and M21 and their subsequent metabolites from JP-1366 compared with the case of anti-CYP1A2, anti-CYP2C19, and anti-CYP2D6 antibodies (Figure 8) in HLMs, supporting the idea that CYP3A4 and CYP3A5 may play major roles in the metabolism of JP-1366. These properties are comparable to those of other P-CAB drugs.

The major involvement of CYP3A4 is a common feature; however, P-CAB drugs displayed different chemical structures, which exhibited different pharmacological features and metabolic fates. Vonoprazan (TAK-438), a sulfonyl pyrrole derivative with a pKa value of 9.37, was primarily metabolized by CYP3A4 to form M-I (5-(2-fluorophenyl)-1-(pyridin-3-ylsulfonyl)-1H-pyrrole-3-carboxylic acid), M-III ([5-(2-fluorophenyl)-1-(pyridin-3-ylsulfonyl)-1H-pyrrol-3-yl]methylidene-N-methylamine oxide), and *N*-demethylated TAK-438, and it was partly metabolized via CYP2B6 to M-I, M-III, and N-demethylated TAK-438; CYP2C19 to M-I and N-demethylated TAK-438; and CYP2D6 to M-III and N-demethylated TAK-438 and sulfotransferase 2A1 [34,35]. Multiple CYP enzymes are involved in the formation of major metabolites, which may reduce CYP2C19-mediated drug interactions and genetic polymorphisms.

Fexuprazan, a sulfonyl pyrrole derivative similar to vonoprazan, was primarily metabolized to M14 via oxidative deamination and M11 via hydroxylation mediated by CYP3A4 with a major contribution of more than 80% and 60%, respectively. CYP2B6, CYP2C19, and CYP2D6 were also involved in the formation of M14 and M11. Major metabolites M14 and M11 did not show an inhibitory effect on H^+^/K^+^-ATPase [36]. Fexuprazan showed the rapid, sustained, and dose-dependent suppression of gastric acid secretion for 24 h after both single and multiple oral administrations. The pharmacokinetics of fexuprazan were not linear after a single dose, but following multiple doses, plasma concentrations increased in a dose-proportional manner, without signs of plasma accumulation. The drug was well tolerated, with no signs of hepatotoxicity, mainly because fexuprazan does not have the imidazopyridine structure that has been known as hepatotoxic [37,38]. According to a comparative study of fexuprazan and esomeprazole to assess their efficacy and safety in patients with erosive esophagitis, fexuprazan 40 mg is non-inferior to esomeprazole 40 mg in erosive esophagitis healing upon 8-week treatment [39].

Tegoprazan, a benzimidazole-6-carboxamide derivative with a pKa value of 5.2, was also primarily metabolized via CYP3A4 and CYP2C19 to the active metabolite M1 with a greater contribution of CYP3A4 (about 75%) [33]. In a preclinical study, tegoprazan metabolite M1 showed reversible inhibitory potential against porcine H^+^/K^+^-ATPase, with 10-fold less potency than tegoprazan [33,40]. IC_50_ values for tegoprazan and M1 were 0.53 μM and 6.19 μM, respectively. Based on preclinical studies and in vitro assay results, M1 is also expected to show an acid suppression effect [33,41]. As a coordinated response of tegoprazan and active metabolite M1, the % time pH > 4 over a 24 h period was 48.9% after a single oral dose of 50 mg of tegoprazan in six healthy subjects and 70.4% after a repeated oral dose of 100 mg of tegoprazan [42]. However, repeated administrations of 100 mg of tegoprazan once daily decreased C_max_ by 40% compared with a single dose (C_max_: 1413 ± 24.7 ng/mL at day 1 vs. 845.2 ± 40.7 ng/mL at day 7) [42] because of the decrease in the acidity of gastric fluid and its pH-dependent absorption [33,41].

JP-1366, an imidazol[1,2-a]pyridine derivative, has a structure similar to tegoprazan, and its major metabolite M1 also had a partial activity. M1 showed an inhibitory effect on H^+^/K^+^-ATPase in a concentration-dependent manner, and the IC_50_ values of JP-1366 and M1 for the H^+^/K^+^-ATPase inhibition were 21.6 nM and 66.5 nM, respectively [1]. The elimination half-life of JP-1366 was 6–9 h in a single oral dose of 5–60 mg in healthy male subjects, and dose linearity was observed without evidence of accumulation in plasma. Therefore, C_max_ and AUC values of JP-1366 were not changed whether it was administered in a single dose or repeated doses for 7 days. The % time pH > 4 over a 24 h period was correlated with AUC values or administered dose (5–40 mg) of JP-1366 in the SAD study, and the values were maintained in the MAD study. In all cases, the % time pH > 4 at nighttime was significantly higher in the repeated JP-1366 dose (40 mg) group (89.28% ± 9.12%) compared to those of the esomeprazole 40 mg dose group (56.56% ± 22.47%), which indicated that JP-1366 had a stronger acid suppression compared to esomeprazole at nighttime. The active metabolite M1 could have a beneficial effect on the stronger and prolonged efficacy of JP-1366. Therefore, the analysis of plasma concentrations of active metabolite M1 as well as JP-1366 seemed to be essential in the clinical studies of pharmacokinetics and the drug response of JP-1366. In addition, % time pH > 4 over a 24 h period of 40 mg of JP-1366 dose was 89.28 ± 9.12% [1] while 50 mg of tegoprazan dose reported 66.0 ± 15.7% of % time pH > 4 over a 24 h period in other clinical study [11]. In a nonclinical efficacy study, JP-1366 has the fastest-acting and superior gastric acid secretion inhibitory effect among the tested drugs (JP-1366, vonoprazan, and tegoprazan) in a lumen-perfused rat model [43]. JP-1366 showed an excellent inhibition of histamine-stimulated gastric acid secretion in the Heidenhain pouch dog model, which was equal to or higher than vonoprazan [39]. It also showed greater GERD lesion inhibition rates in rats compared with vonoprazan [43]. Comparisons of the efficacy of JP-1366 with other PPIs and P-CAB drugs need to be performed in the same clinical study.

Despite the fact that extensive pharmacokinetic studies regarding the target tissue distribution and elimination pathway still need to be conducted, JP-1366 is a highly permeable drug in Caco-2 cells (Table 7) and demonstrates high HER (Figure 1). CYP3A4 and CYP3A5 are the primary metabolizing enzymes that mediate the formation of the major metabolites of JP-1366 (i.e., *N*-dearylation and hydroxylation). However, the involvement of multiple metabolizing enzymes such as CYP1A2, CYP2C9/19, CYP1A2, and CYP2D6 with a minor contribution in the formation of M1, M6, M19, and M21 and the involvement of UGT1A1 and UGT2B7 in the formation of M15 from M1 and the formation of 23 metabolites might reduce the risk of drug–drug interaction potential mediated by a single metabolizing enzyme and a main metabolite. Additionally, it may reduce the intersubject variability caused by genetic variations in these metabolizing enzymes. In accordance with this, the dose-normalized AUC of JP-1366 was not significantly different among the selected genetic variants of CYP2C19, CYP3A5, CYP2D6, UGT1A1, and UGT2B17 that are involved in the metabolism of JP-1366 [1]. The lack of involvement of JP-1366 in the clinically relevant drug transporters also reduces the risk of transporter-mediated drug–drug interaction potential. JP-1366 did not show substrate specificity on uptake transporters that govern the tissue-selective distribution and elimination nor on efflux transporters such as P-gp and BCRP (Table 6). To understand the off-target risk of JP-1366, the molecular target search for JP-1366 (10 μM) through BioPrint^®^ profiling (Eurofins CEREP SA, Celle-Lévescault, France) was conducted. Among them, four targets (A3, α2B, Cl^-^ channel, and MT1) with high inhibition rates by JP-1366 were found with IC_50_ values of 2.7, 1.2, 0.91, and 0.87 μM, respectively. The IC_50_ values are 44- to 137-fold higher than IC_50_ (19.7 nM) for H^+^/K^+^-ATPase inhibition, suggesting that JP-1366 selectively acts on H^+^/K^+^-ATPase inhibition. 

## 5. Conclusions

JP-1366 (zastaprazan) is highly permeable in intestinal absorption and undergoes high hepatic extraction. It was metabolized to 23 metabolites (18 phase I metabolites and 5 phase II metabolites) in human hepatocytes. Phase I metabolism contains *N*-dearylation (M6), *N*-dearylation and hydroxylation (monohydroxylation, M3, M4; di-, M5; tri-, M2), hydroxylation (mono-, M1, M19, M21; di-, M7, M8, M14, M22; tri-, M13, M18), hydroxylation and reduction (mono-, M20; di-, M9, M16), and hydrolysis (M23). Phase II metabolism contains hydroxylation and glucuronidation (M11, M15), hydroxylation and sulfation (M17), and dihydroxylation and sulfation (M10, M12). CYP3A4 and 3A5 played major roles in the formation of most phase I metabolites except for M3, M4, M5, M2, M20, and M23, and UGT2B7 and 2B17 were responsible for the glucuronidation of M1 to M15. In contrast to the case of drug-metabolizing enzymes, drug transporters played limited roles in the uptake or efflux of JP-1366 and active metabolite M1. Only M1 is a substrate for P-gp. Therefore, further pharmacokinetic interactions and pharmacogenetic studies need to be conducted considering the enzymes involved in its metabolism and P-gp.

## Figures and Tables

**Table 1:** MS/MS parameters for the detection of JP-1366 and metabolites.

Analytes	Mass Transition (*m*/*z*)	Collision Energy (V)	t_R_
JP-1366	363.1 → 119.1	20	26.6
M1	379.2 → 107.1	24	19.1
M2	293.1 → 233.0	32	0.9
M3	261.1 → 233.0	28	1.2
M4	261.1 → 243.0	28	1.3
M5	277.1 → 217.1	28	1.7
M6	245.1 → 217.1	28	2.6
M7	395.0 → 150.9	22	9.0
M8	395.0 → 107.1	24	15.8
M13	411.0 → 107.0	20	16.9
M14	395.0 → 135.0	20	17.0
M16	397.0 → 119.0	20	19.6
M18	411.0 → 119.0	20	20.2
M19	379.0 → 135.0	24	20.4
M20	381.0 → 119.0	20	22.5
M21	379.2 → 119.1	20	23.4
M22	395.0 → 119.0	20	23.3
M23	324.0 → 119.0	20	24.0
Lansoprazole (IS)	369.9 → 252.0	10	2.74

**Table 2:** Retention time (t_R_), molecular ion ([M+H]^+^), empirical formula, and product ions of JP-1366 and its possible metabolites after incubation with human (H), dog (D), monkey (Mk), mouse (M), and rat (R) hepatocytes.

Name	t_R_ (min)	[M+H]^+^ (*m*/*z*)	Formula	Product Ions	Hepatocytes
JP-1366	27.58	363.21794	C_22_H_26_N_4_O	119.08553, 188.08184, 245.13969, 257.13969, 306.16009	
M1	20.33	379.21285	C_22_H_26_N_4_O_2_	107.08553, 135.08044, 188.08184, 245.13969, 304.14444, 361.20229	H, D, Mk, M, R
M2	1.16	293.12443	C_13_H_16_N_4_O_4_	203.09274, 221.10330, 233.10330, 247.11895, 263.11387, 275.11387	H, D, Mk, M
M3	1.42	261.13460	C_13_H_16_N_4_O_2_	203.09274, 215.09274, 233.10330, 243.12404	H, D, Mk, M, R
M4	1.55	261.13460	C_13_H_16_N_4_O_2_	203.09274, 215.09274, 233.10330, 243.12404	H, D, Mk, R
M5	2.21	277.12952	C_13_H_16_N_4_O_3_	193.08458, 205.10839, 217.10839	H, D, Mk, M, R
M6	3.22	245.13969	C_13_H_16_N_4_O	161.09475, 188.08184, 217.10839	H, D, Mk, M, R
M7	10.40	395.20777	C_22_H_26_N_4_O_3_	151.07536, 188.08184, 245.13969, 320.13935, 359.18664, 377.19720	H, Mk, M, R
M8	17.01	395.20777	C_22_H_26_N_4_O_3_	107.08553, 135.08044, 204.07675, 261.13460, 320.13935, 377.19720	H, D, Mk, M, R
M9	17.52	397.22342	C_22_H_28_N_4_O_3_	107.08553, 135.08044, 188.08184, 263.15025, 304.14444, 322.15500, 379.21285	H, D, Mk, M, R
M10	17.64	475.16459	C_22_H_26_N_4_O_6_S	135.08044, 215.03726, 243.12404, 261.13460, 273.13460, 338.14992, 377.19720, 395.20777	H, D, Mk, M, R
M11	17.81	555.24494	C_28_H_34_N_4_O_8_	107.08553, 135.08044, 245.13969, 257.13969, 311.11253, 379.21285	H, Mk, M, R
M12	17.96	475.16459	C_22_H_26_N_4_O_6_S	135.08044, 215.03726, 243.12404, 261.13460, 273.13460, 338.14992, 377.19720, 395.20777	H, D, Mk, M, R
M13	18.02	411.20268	C_22_H_26_N_4_O_4_	107.08553, 135.08044, 188.08184, 277.12952, 333.17099, 393.19212	H, D, Mk, M, R
M14	18.18	395.20777	C_22_H_26_N_4_O_3_	135.08044, 204.07675, 243.12404, 261.13460, 338.14992	H, D, Mk, M, R
M15	19.11	555.24494	C_28_H_34_N_4_O_8_	135.08044, 245.13969, 304.14444, 361.20229, 379.21285	H, D, Mk, M, R
M16	20.78	397.22342	C_22_H_28_N_4_O_3_	119.08553, 204.07675, 261.13460, 291.14517, 367.21285	H, D, Mk, M, R
M17	21.20	459.16967	C_22_H_26_N_4_O_5_S	135.08044, 188.08184, 215.03726, 245.13969, 257.13969, 322.15500, 379.21285	H, D, Mk, M, R
M18	21.50	411.20268	C_22_H_26_N_4_O_4_	119.08553, 245.10335, 275.11387, 305.12443, 381.19212	H, D, Mk, M
M19	21.65	379.21285	C_22_H_26_N_4_O_2_	135.08044, 188.08184, 245.13969, 257.13969, 322.15500	H, D, Mk, M, R
M20	23.69	381.22850	C_22_H_28_N_4_O_2_	119.08553, 188.08184, 275.15025, 306.16009	H, D, Mk, M, R
M21	24.42	379.21285	C_22_H_26_N_4_O_2_	119.08553, 204.07675, 243.12404, 261.13460, 273.13460, 349.20229	H, D, Mk, M, R
M22	24.43	395.20777	C_22_H_26_N_4_O_3_	119.08553, 188.08184, 229.10839, 277.12952, 289.12952	H, D, Mk, M, R
M23	25.15	324.17065	C_19_H_21_N_3_O_2_	119.08553, 206.09240	H, D, M, R

**Table 3:** Enzyme kinetic parameters for the metabolism of JP-1366 in human liver microsomes (HLM) and cDNA-expressed CYP isozymes.

Parameters	CYP1A2	CYP2C8	CYP2C9	CYP2C19	CYP2D6	CYP3A4	CYP3A5	HLM
M1								
*K*_m_ (μM)	32.4	12.4	2.0	4.0	-	13.2	19.2	17.0
*V*_max_	0.046	0.38	0.29	0.27	-	4.6	6.3	826.1
*K*_i_ (μM)	-	197.3	-	-	-	-	-	96.5
*CL*_int_	0.0014	0.031	0.14	0.068	-	0.35	0.33	48.6
*n*	0.6	-	1.2	1.6	-	2.9	1.5	-
Contribution (%)	0.1	3.7	10.3	0.1	-	17.3	68.6	
M3 ^b^								
*K*_m_ (μM)	-	-	-	-	-	12.5	2.4	6.8
*V*_max_	-	-	-	-	-	2.1	0.18	53.3
*K*_i_ (μM)	-	-	-	-	-	29.3	-	32.2
*CL*_int_	-	-	-	-	-	0.17	0.076	7.8
*n*	-	-	-	-	-	-	1.6	-
Contribution (%)						79.9	20.1	
M6								
*K*_m_ (μM)	-	-	9.6	111.0	158.4	8.5	13.3	14.3
*V*_max_	-	-	0.068	0.25	0.24	6.5	5.5	798.8
*K*_i_ (μM)	-	-	-	-	-	-	-	104.6
*CL*_int_	-	-	0.0070	0.0023	0.0015	0.77	0.41	55.9
*n*	-	-	0.7	0.6	0.7	1.8	1.5	-
Contribution (%)			2.8	0.1	0.1	28.1	68.9	
M8 ^a^								
*K*_m_ (μM)	-	-	-	-	-	16.7	4.2	5.2
*V*_max_	-	-	-	-	-	0.75	0.18	12.8
*K*_i_ (μM)	-	-	-	-	-	32.0	-	5.3
*CL*_int_	-	-	-	-	-	0.045	0.044	2.5
*n*	-	-	-	-	-	-	1.3	-
Contribution (%)						58.3	41.7	
M14 ^c^								
*K*_m_ (μM)	-	-	-	-	-	41.8	6.1	3.9
*V*_max_	-	-	-	-	-	0.16	0.036	1.9
*K*_i_ (μM)	-	-	-	-	-	22.9	187.0	102.0
*CL*_int_	-	-	-	-	-	0.0037	0.0060	0.50
Contribution (%)						59.2	40.8	
M16 ^c^								
*K*_m_ (μM)	-	-	-	-	5.4	8.1	7.0	4.9
*V*_max_	-	-	-	-	0.026	0.050	0.016	2.5
*CL*_int_	-	-	-	-	0.0048	0.0062	0.0023	0.51
*n*	-	-	-	-	1.9	2.4	1.3	17.4
Contribution (%)					3.3	49.7	47.1	
M19 ^c^								
*K*_m_ (μM)	12.4	5.0	3.4	4.1	7.5	15.9	20.1	8.2
*V*_max_	0.014	0.16	0.036	0.056	0.028	0.64	1.5	85.1
*CL*_int_	0.0011	0.032	0.011	0.014	0.0037	0.040	0.074	10.3
*n*	0.7	1.4	1.2	1.7	2.9	3.2	1.6	1.5
Contribution (%)	0.2	7.3	6.0	0.1	0.1	11.0	75.5	
M20 ^c^								
*K*_m_ (μM)	22.5	-	-	1091.7	5.1	-	-	22.4
*V*_max_	0.021	-	-	0.10	0.96	-	-	64.9
*CL*_int_	0.0010	-	-	0.0001	0.19	-	-	2.9
*n*	0.6	-	-	0.3	1.7	-	-	1.6
Contribution (%)	9.8			3.8	86.4			
M21 ^c^								
*K*_m_ (μM)	16.6	7.0	2.3	4.8	-	17.4	18.9	8.8
*V*_max_	1.67	0.20	0.039	0.26	-	3.2	2.1	342.4
*CL*_int_	0.10	0.028	0.017	0.054	-	0.18	0.11	38.9
*n*	0.9	1.2	1.2	1.4	-	3.0	1.6	1.6
Contribution (%)	9.5	4.4	3.3	0.1	-	27.9	54.8	
M22 ^c^								
*K*_m_ (μM)	-	-	-	4.2	6.5	9.5	11.9	8.6
*V*_max_	-	-	-	0.019	0.43	0.37	0.13	25.7
*CL*_int_	-	-	-	0.0045	0.067	0.039	0.011	3.0
*n*	-	-	-	0.9	1.6	2.2	1.4	1.5
Contribution (%)				0.1	6.8	45.2	47.9	
M23 ^c^								
*K*_m_ (μM)	-	-	-	-	-	-	-	17.4
*V*_max_	-	-	-	-	-	-	-	23.2
*CL*_int_	-	-	-	-	-	-	-	1.3

*V*_max_: pmol/min/pmol CYP for CYP isozymes and pmol/min/mg protein for human liver microsomes; *CL*_int_: μL/min/pmol CYP isozymes and μL/min/mg protein for human liver microsomes; *n*: Hill coefficient; -: not calculated; ^a^: quantification via M1 standard curve; ^b^: quantification via M6 standard curve; ^c^: quantification via JP-1366 standard curve.

**Table 4:** Enzyme kinetic parameters for the formation of M15 from M1 in human liver microsomes (HLM) and human cDNA-expressed UGT 2B7 and 2B17 isozymes.

System	*K_m_* (μM)	*V_max_* (pmol/min/mg Protein)	*K_i_* (μM)	*CL_int_* (μL/min/mg Protein)	*n*
HLM	57.9	74.9	-	1.29	1.0
UGT2B7	178.8	107.1	-	0.60	0.8
UGT2B17	6.5	8.27	59.0	1.27	-

*n*: Hill coefficient; -: not calculated.

**Table 5:** Uptake of 6 μM of JP-1366 and M1 into HEK293 cells expressing MATE1, MATE2K, OCT1, OCT2, OAT1, OAT3, OATP1B1, and OATP1B3 transporters.

Compounds	Cells	Treatment	Uptake Rate (pmol/min/10^5^ Cells)	Fold-Change	Substrate
JP-1366	HEK293-mock	control	51.53 ± 3.16	1.0	
		cimetidine 100 μM	55.35 ± 2.24	1.1	
		probenecid 20 μM	54.22 ± 1.90	1.1	
		rifampin 20 μM	57.80 ± 0.89	1.1	
	HEK293-MATE1	control	65.32 ± 4.13	1.3	No
		cimetidine 100 μM	78.57 ± 5.38	1.5	
	HEK293-MATE2K	control	59.19 ± 3.66	1.1	No
		cimetidine 100 μM	72.81 ± 3.33	1.4	
	HEK293-OCT1	control	82.43 ± 3.59	1.6	No
		cimetidine 100 μM	82.01 ± 3.72	1.6	
	HEK293-OCT2	control	51.71 ± 1.36	1.0	No
		cimetidine 100 μM	49.28 ± 4.50	1.0	
	HEK293-OAT1	control	68.04 ± 2.96	1.3	No
		probenecid 20 μM	61.04 ± 5.29	1.2	
	HEK293-OAT3	control	58.53 ± 3.71	1.1	No
		probenecid 20 μM	52.47 ± 2.27	1.0	
	HEK293-OATP1B1	control	52.79 ± 2.39	1.0	No
		rifampin 20 μM	53.60 ± 3.73	1.0	
	HEK293-OATP1B3	control	44.54 ± 0.74	0.9	No
		rifampin 20 μM	52.67 ± 2.99	1.0	
M1	HEK293-mock	control	51.53 ± 3.16	1.0	
		cimetidine 100 μM	55.35 ± 2.24	1.1	
		probenecid 20 μM	54.22 ± 1.90	1.1	
		rifampin 20 μM	57.80 ± 0.89	1.1	
	HEK293-MATE1	control	65.32 ± 4.13	1.3	No
		cimetidine 100 μM	78.57 ± 5.38	1.5	
	HEK293-MATE2K	control	59.19 ± 3.66	1.1	No
		cimetidine 100 μM	72.81 ± 3.33	1.4	
	HEK293-OCT1	control	82.43 ± 3.59	1.6	No
		cimetidine 100 μM	82.01 ± 3.72	1.6	
	HEK293-OCT2	control	51.71 ± 1.36	1.0	No
		cimetidine 100 μM	49.28 ± 4.50	1.0	
	HEK293-OAT1	control	68.04 ± 2.96	1.3	No
		probenecid 20 μM	61.04 ± 5.29	1.2	
	HEK293-OAT3	control	58.53 ± 3.71	1.1	No
		probenecid 20 μM	52.47 ± 2.27	1.0	
	HEK293-OATP1B1	control	52.79 ± 2.39	1.0	No
		rifampin 20 μM	53.60 ± 3.73	1.0	
	HEK293-OATP1B3	control	44.54 ± 0.74	0.9	No
		rifampin 20 μM	52.67 ± 2.99	1.0	

Each datapoint represents the mean ± SD (*n* = 3). Fold-change was calculated by dividing the mean uptake rate of JP-1366 or M1 in the treatment group in HEK293-overexpressed cells by the mean uptake rate of JP-1366 or M1 in the corresponding treatment group in HEK293-mock cells.

**Table 6:** Permeability of 4 μM of JP-1366 and M1 in LLC-PK1-P-gp and LLC-PK1-BCRP cells.

Compounds	Cells	Treatment	P_app_ (×10^−6^ cm/s) ^a^		ER ^b^	Substrate
			A to B	B to A		
JP-1366	LLC-PK1-P-gp	Control	20.28 ± 0.53	25.65 ± 1.71	1.3	No
		CsA 20 μM	20.69 ± 1.31	32.05 ± 0.94	1.5	
	LLC-PK1-BCRP	Control	14.84 ± 1.23	20.52 ± 3.95	1.4	No
		Ko134 1 μM	13.58 ± 0.54	22.62 ± 0.47	1.7	
M1	LLC-PK1-P-gp	Control	6.99 ± 0.60	99.30 ± 1.66	14.2	Yes
		CsA 20 μM	18.52 ± 0.96	62.83 ± 5.26	3.4	
	LLC-PK1-BCRP	Control	31.66 ± 3.39	33.80 ± 3.45	1.4	No
		Ko134 1 μM	31.97 ± 2.84	47.31 ± 1.39	1.7	

^a^ The mean ± SD (*n* = 3); ^b^ by dividing mean P_app,BA_ by mean P_app,AB_.

**Table 7:** Apparent permeability (P_app_) in Caco-2 cells.

Group	Compound	P_app_ (×10^−6^ cm/s) ^a^		ER ^b^
		A to B	B to A	
Marker	Caffeine	27.00 ± 1.55	26.05 ± 1.95	0.96
	Propranolol	9.75 ± 0.68	12.20 ± 1.51	1.3
	Ofloxacin	3.45 ± 0.03	4.79 ± 0.15	1.4
	Atenolol	0.11 ± 0.01	0.11 ± 0.01	0.98
Test compounds	JP-1366	22.98 ± 5.79	10.70 ± 1.77	0.47
	M1	3.36 ± 0.24	20.30 ± 1.51	6.0

^a^ The mean ± SD (*n* = 3); ^b^ by dividing mean P_app,BA_ by mean P_app,AB_.

## Data Availability

Data are contained within the article and Supplementary Materials.

## Supplementary Materials

The following supporting information can be downloaded at https://www.mdpi.com/article/10.3390/pharmaceutics16060799/s1: Figure S1: MS/MS spectrum and possible fragmentation pattern of (A) M2, (B) M5, (C) M3, and (D) M4; Figure S2: MS/MS spectrum and possible fragmentation pattern of (A) M7, (B) M8, (C) M9, (D) M13, and (E) M15; Figure S3: MS/MS spectrum and possible fragmentation pattern of (A) M19, (B) M11, (C) M17, (D) M14, (E) M10, and (F) M12; Figure S4: MS/MS spectrum and possible fragmentation pattern of (A) M21, (B) M16, (C) M20, (D) M22, and (E) M18; Figure S5: Concentration-dependent formation rates of JP-1366 metabolites in human cDNA-expressed CYP3A4 isozymes in the concentration range of 1–80 μM of JP-1366. Insets are Eadie-Hofstee plots. Each data represents mean ± SD (*n* = 3).
