# Supplementary material for: In Vitro Metabolism and Transport Characteristics of Zastaprazan
Source: Pharmaceutics. 2024 Jun 13;16(6):799. doi: 10.3390/pharmaceutics16060799 (PMC11207335; doi:10.3390/pharmaceutics16060799)
Supplement: Supplementary file 1 [file pharmaceutics-16-00799-s001.zip › pharmaceutics-3053816-supplementary.pdf]

Article

In vitro metabolism and transport characteristics of zastaprazan

Min Seo Lee[1,#], Jihoon Lee[2,#], Minyoung Pang[3], John Kim[4], Hyunju Cha[4], Banyoon Cheon[4], Min Koo Choi[3], Im-Sook Song[2,*], Hye Suk Lee [1,*]

1 College of Pharmacy and BK21 Four-sponsored Advanced Program for SmartPharma Leaders, The Catholic University of Korea, Bucheon 14662, Republic of Korea. minseo.lee@catholic.ac.kr (M.S.L.); sianalee@catholic.ac.kr (L.H.S.)

2 BK21 FOUR Community-Based Intelligent Novel Drug Discovery Education Unit, Vessel-Organ Interaction Research Center (VOICE), Research Institute of Pharmaceutical Sciences, College of Pharmacy, Kyungpook National University, Daegu 41566, Republic of Korea. legadema0905@knu.ac.kr (J.L.); isssong@knu.ac.kr (I.-S.S.)

3 College of Pharmacy, Dankook University, Cheonan 30019, Republic of Korea. mignon@dankook.ac.kr (M.P.); minkoochoi@dankook.ac.kr (M.-K.C.)

4 Onconic Therapeutics Inc, Seoul 06236, Republic of Korea. john.kim@onconic.co.kr (J.K.); hj.cha@onconic.co.kr (H.C.); bycheon@onconic.co.kr (B.C.)

[#] These authors equally contribute this work.

* Correspondence: sianalee@catholic.ac.kr (H.S.L.), isssong@knu.ac.kr (I.S.S.)

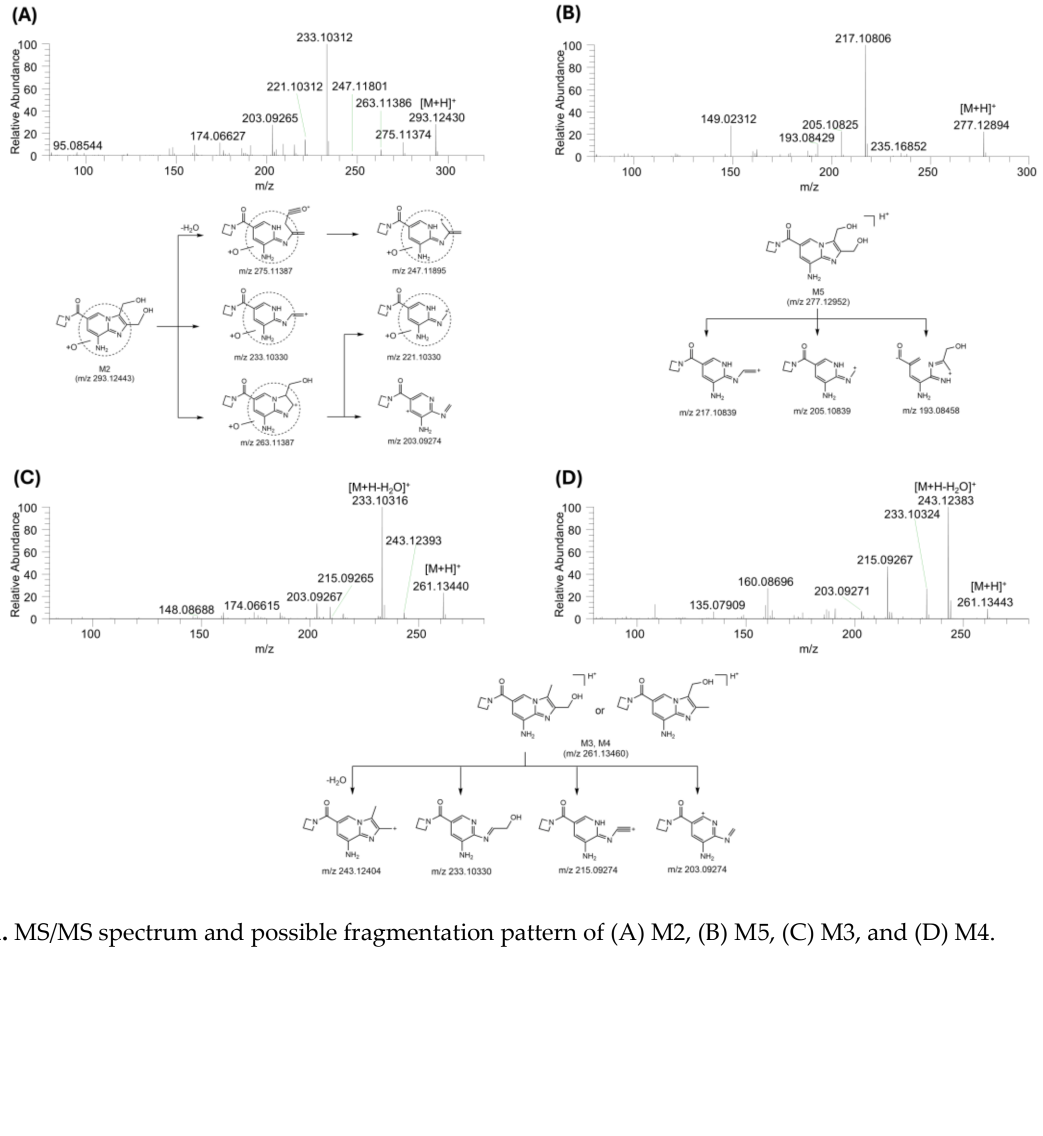

Figure S1. MS/MS spectrum and possible fragmentation pattern of (A) M2, (B) M5, (C) M3, and (D) M4.

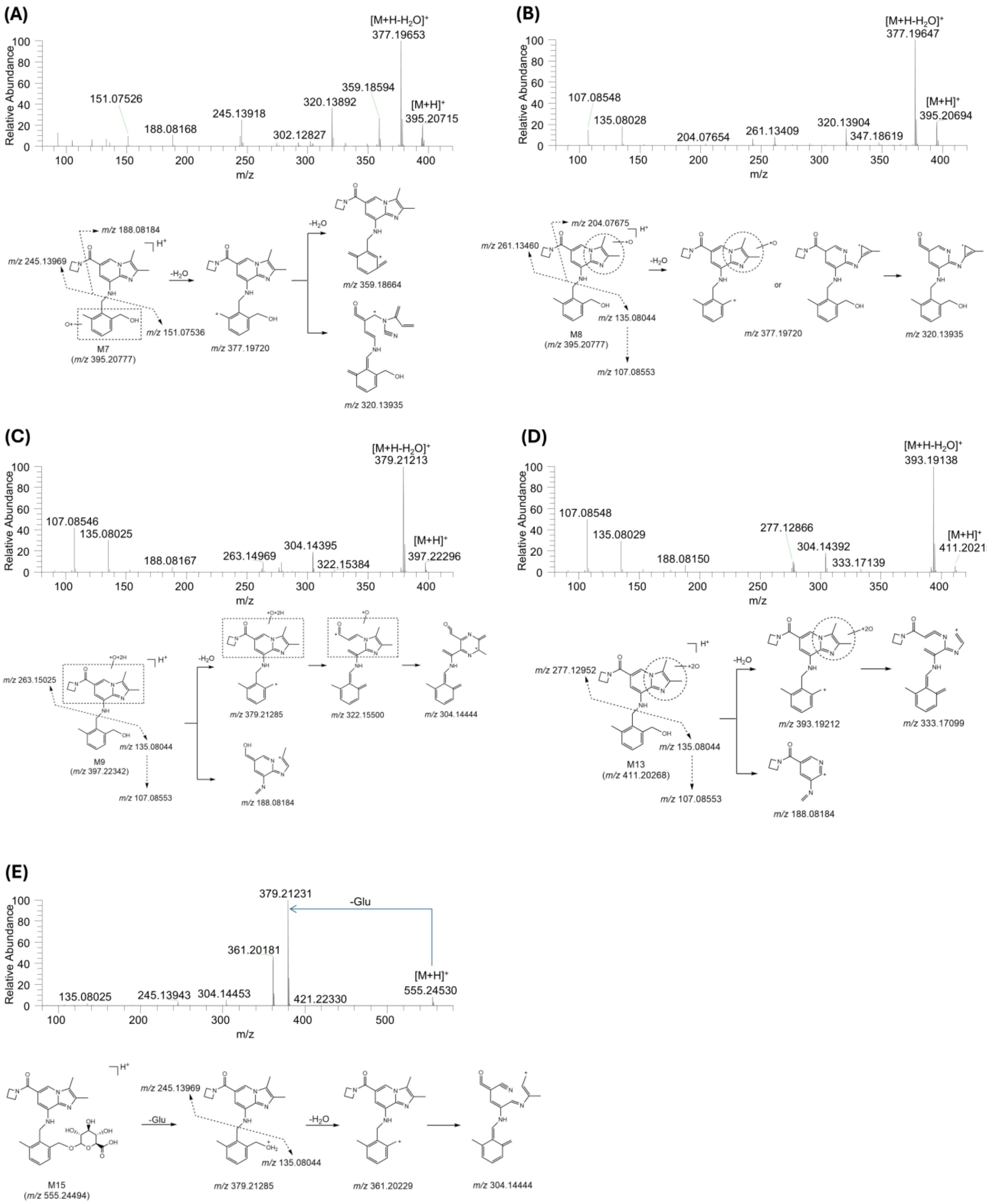

Figure S2. MS/MS spectrum and possible fragmentation pattern of (A) M7, (B) M8, (C) M9, (D) M13, and (E) M15.

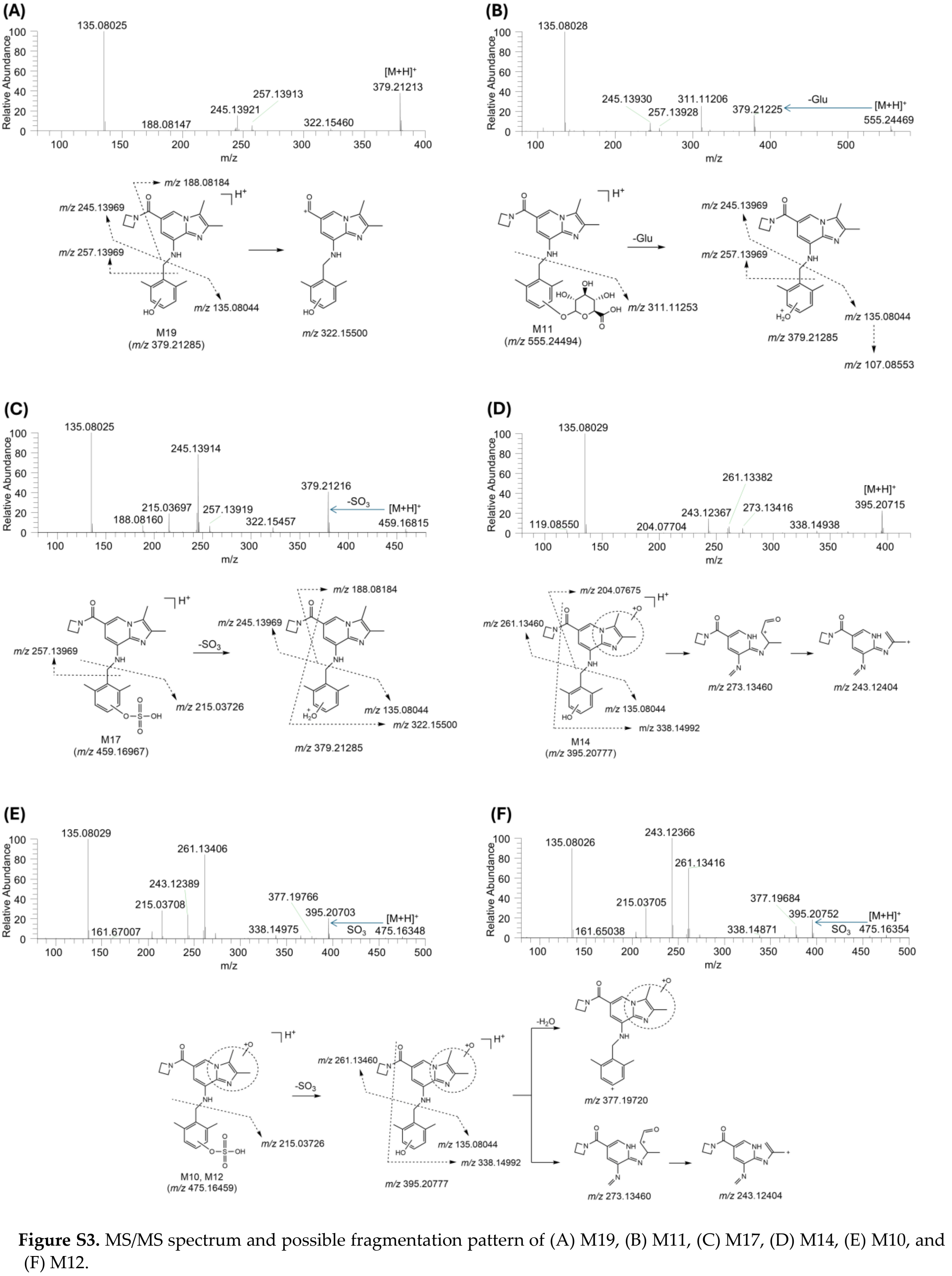

Figure S3. MS/MS spectrum and possible fragmentation pattern of (A) M19, (B) M11, (C) M17, (D) M14, (E) M10, and (F) M12.

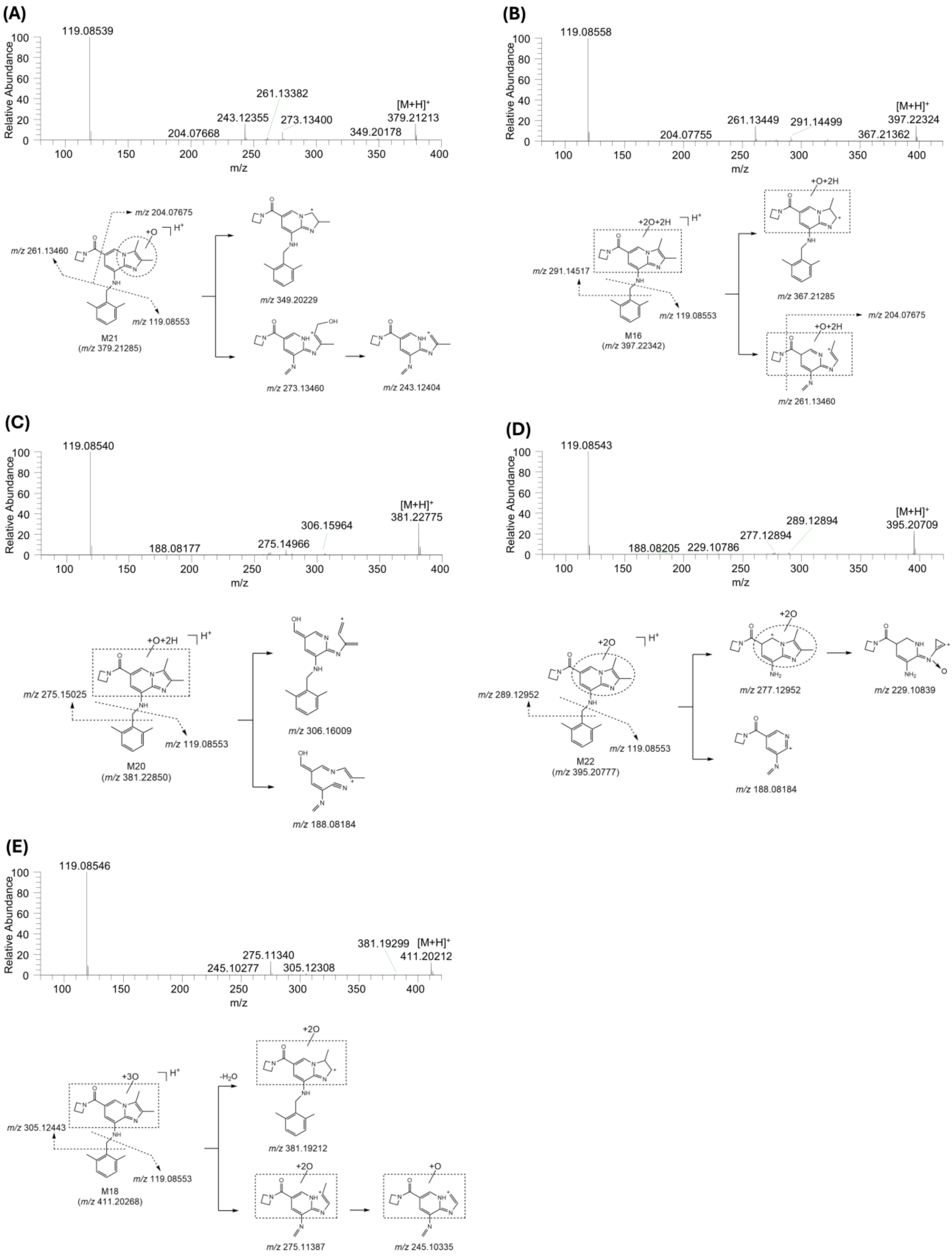

Figure S4. MS/MS spectrum and possible fragmentation pattern of (A) M21, (B) M16, (C) M20, (D) M22, and (E) M18.

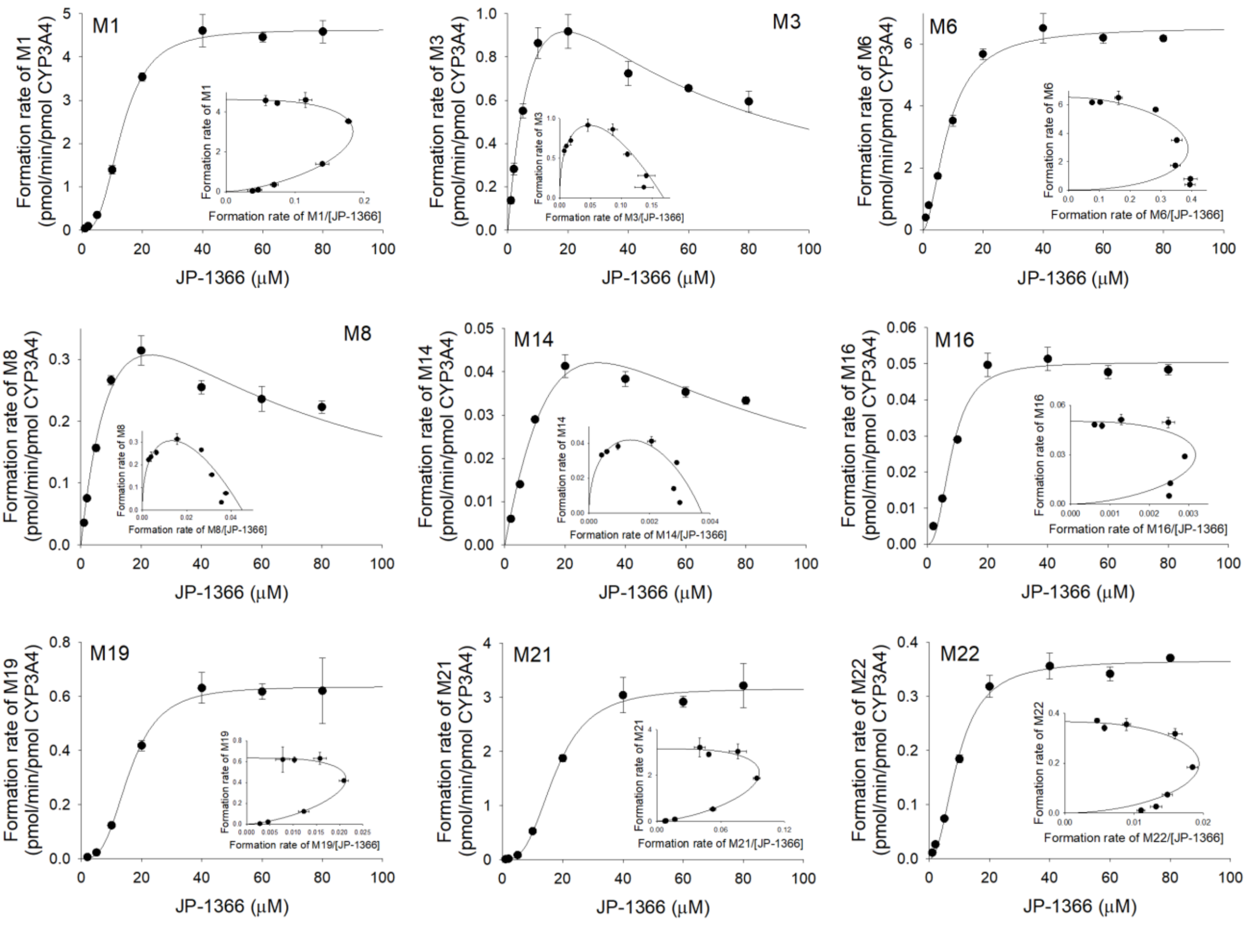

Figure S5. Concentration dependent formation rates of JP-1366 metabolites in human cDNA-expressed CYP3A4 isozymes in the concentration range of 1 – 80 μM of JP-1366. Insets are Eadie-Hofstee plots. Each data represents mean ± SD (n=3).
